# In Vivo Induction of Regulatory T Cells Via CTLA‐4 Signaling Peptide to Control Autoimmune Encephalomyelitis and Prevent Disease Relapse

**DOI:** 10.1002/advs.202004973

**Published:** 2021-05-05

**Authors:** Gil‐Ran Kim, Won‐Ju Kim, Sangho Lim, Hong‐Gyun Lee, Ja‐Hyun Koo, Kyung‐Ho Nam, Sung‐Min Kim, Sung‐Dong Park, Je‐Min Choi

**Affiliations:** ^1^ Department of Life Science College of Natural Sciences Hanyang University Research institute for Natural Sciences Hanyang University Seoul 04763 Republic of Korea; ^2^ Hubrecht Institute for Developmental Biology and Stem Cell Research‐KNAW, University Medical Centre Utrecht Utrecht 3584 CT Netherland; ^3^ Department of Neurology College of Medicine Seoul National University Seoul National University Hospital Seoul 03080 Republic of Korea; ^4^ Department of Life Science College of Natural Sciences Hanyang University Research institute for Natural Sciences Research Institute for Convergence of Basic Sciences Hanyang University Seoul 04763 Republic of Korea

**Keywords:** autoimmunity, CTLA‐4, EAE (experimental autoimmune encephalomyelitis), multiple sclerosis, regulatory T cells

## Abstract

Regulatory T cells play a key role in immune tolerance to self‐antigens, thereby preventing autoimmune diseases. However, no drugs targeting Treg cells have been approved for clinical trials yet. Here, a chimeric peptide is generated by conjugation of the cytoplasmic domain of CTLA‐4 (ctCTLA‐4) with dNP2 for intracellular delivery, dNP2‐ctCTLA‐4, and evaluated Foxp3 expression during Th0, Th1, Treg, and Th17 differentiation dependent on TGF‐*β*. The lysine motif of ctCTLA‐4, not tyrosine motif, is required for Foxp3 expression for Treg induction and amelioration of experimental autoimmune encephalomyelitis (EAE). Transcriptome analysis reveals that dNP2‐ctCTLA‐4‐treated T cells express Treg transcriptomic patterns with properties of suppressive functions. In addition, the molecular interaction between the lysine motif of ctCTLA‐4 and PKC‐*η* is critical for Foxp3 expression. Although both CTLA‐4‐Ig and dNP2‐ctCTLA‐4 treatment in vivo ameliorated EAE progression, only dNP2‐ctCTLA‐4 requires Treg cells for inhibition of disease progression and prevention of relapse. Furthermore, the CTLA‐4 signaling peptide is able to induce human Tregs in vitro and in vivo as well as from peripheral blood mononuclear cells (PBMCs) of multiple sclerosis patients. These results collectively suggest that the chimeric CTLA‐4 signaling peptide can be used for successful induction of regulatory T cells in vivo to control autoimmune diseases, such as multiple sclerosis.

## Graphical abstract

In this study, it is described how the synthetic peptide dNP2‐ctCTLA‐4 can induce Foxp3^+^ Tregs in mice and human peripheral blood mononuclear cells (PBMCs), ameliorate experimental autoimmune encephalomyelitis (EAE) progression with long‐term regulation and prevent disease relapse. On the basis of these findings, this peptide is an attractive drug candidate to increase Tregs in autoimmune diseases, such as multiple sclerosis.

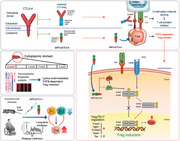

## 1 Introduction

Regulatory T cells are suppressor T cells that express the master transcriptional regulator Foxp3 and CD25 on their surfaces; they also constitutively express cytotoxic T‐lymphocyte‐associated protein 4 (CTLA‐4), with this latter protein critical for the maintenance of immune tolerance and homeostasis.^[^ ^1^ ^]^ Transforming growth factor‐*β* (TGF‐*β*) is a critical cytokine for Foxp3 expression that is required for differentiation of naïve CD4 T cells to Tregs and for maintaining iTreg homeostasis.^[^ ^1^, ^2^ ^]^ Direct transfer of Foxp3^+^ Tregs in vivo reduces or reverses inflammation in inflammatory bowel disease, allergic airway inflammation, and type 1 diabetes.^[^ ^3^ ^]^ Thus, antigen‐specific immune suppression by Tregs is an attractive strategy for regulating excessive immune response.^[^ ^3^ ^]^ Tregs utilize multiple mechanisms for immune regulation, including production of anti‐inflammatory cytokines like IL‐10, deprivation of IL‐2, an important cytokine for proliferation of effector T cells, and transmission of inhibitory signals via CTLA‐4 to both antigen presenting cells (APCs) and T cells.^[^ ^4^ ^]^

CTLA‐4 is an important functional molecule for regulatory T cells (Tregs) and functions as an immune checkpoint protein that negatively regulates T cell activation and contributes peripheral tolerance.^[^ ^4^, ^5^ ^]^ CTLA‐4 shares its ligands with CD28, namely CD80 and CD86 expressed by APCs; however, its avidity for these ligands is greater than that of CD28, and it inhibits costimulatory signaling in T cells.^[^ ^6^ ^]^ In addition, CTLA‐4 has an evolutionarily highly conserved cytoplasmic domain with an immunoreceptor tyrosine‐based inhibitory motif (ITIM) ‐like motif that transmits a negative signal to T cells via recruitment of SH2 domain‐containing protein tyrosine phosphatase (SHP) or PP2A, thereby inhibiting T cell receptor signaling.^[^ ^7^ ^]^ Treg‐specific CTLA‐4 knockout impairs the suppressive function of Foxp3‐expressing Tregs, suggesting that CTLA‐4 is critically required for Treg function.^[^ ^4c^ ^]^ In addition, biochemical analyses have revealed that protein kinase C‐*η* (PKC‐*η*) and CTLA‐4 interaction is needed to reduce CD86 expression by APCs, emphasizing the importance of CTLA‐4 in Treg biology.^[^ ^8^ ^]^

Interestingly, a CTLA‐4 splicing variant lacking the B7 binding domain was still able to control T cell activation, and expression of a B7‐nonbinding CTLA‐4 mutant in CTLA‐4 knockout T cells inhibited T cell proliferation and cytokine production.^[^ ^9^ ^]^ In our previous study, we reported that intracellular delivery of the cytoplasmic domain of CTLA‐4 (ctCTLA‐4) in vivo ameliorated allergic inflammation, autoimmune disease, and graft rejection.^[^ ^10^ ^]^

Although Tregs and CTLA‐4 have been intensively studied as critical targets for controlling immune tolerance in human diseases, no immunomodulatory drugs for in vivo Treg generation have been approved for human disease therapy. Here, we generated a synthetic chimeric peptide comprising the cytoplasmic domain of CTLA‐4 conjugated with the blood brain barrier (BBB)‐permeable peptide dNP2 to stimulate Foxp3 expression in murine and human T cells to treat autoimmune disease. Our evidence suggests that this therapeutic peptide (dNP2‐ctCTLA‐4) can serve as a novel agent to induce Tregs in vivo to regulate excessive immune responses and potentially cure autoimmune diseases.

## 2 Results

### 2.1 Treg Induction by a Synthetic Peptide with the Cytoplasmic Domain of CTLA‐4

**Figure 1:**
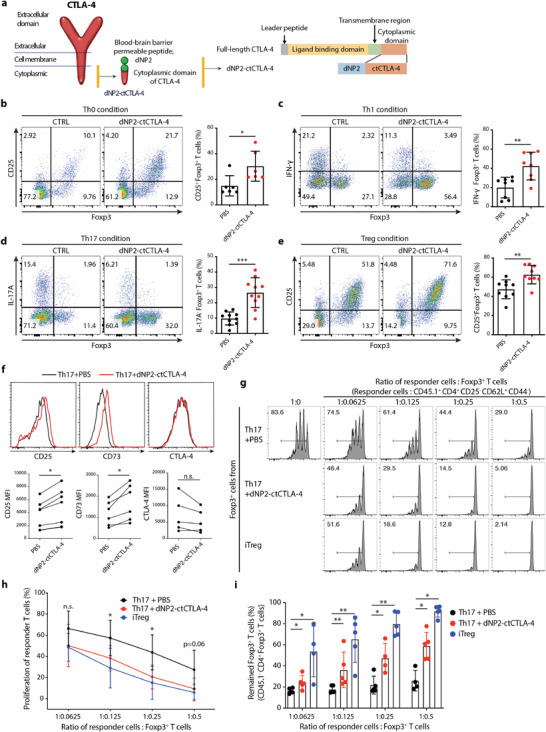
The cytoplasmic domain of the CTLA‐4 peptide induces functional Foxp3^+^ Tregs in vitro. a) Schematic of the synthetic CTLA‐4 signaling peptide. b–e) Mouse naïve CD4 T cells were stimulated with anti‐CD3/CD28 mAb in the presence or absence of 2 × 10^−6^ m dNP2‐ctCTLA‐4 for 4 days. Representative flow cytometry dot plots (left) and bar graphs (right) of Foxp3 induction under b) Th0, c) Th1, d) Th17, or e) iTreg differentiation conditions (*n* = 6–10). f) Treg markers including CD25, CD73, and CTLA‐4 under Th17 differentiation conditions were analyzed by flow cytometry (*n* = 5–7). g–i) In vitro suppression assay based on coculture of Foxp3^+^ T cells (CD45.1^‐^ GFP^+^) and responder T cells (CD45.1^+^ CD4^+^ CD25^−^ CD62L^+^ CD44^−^) for 3 days (*n* = 4–5). Foxp3^+^ T cells induced by 2 × 10^−6^ m of CTLA‐4 signaling peptide were sorted and then cocultured with responder T cells for 3 days. Naïve CD4 responder T cells were isolated from splenocytes, labeled with carboxyfluorescein succinimidyl ester (CFSE), and stimulated with CD3/28 dynabeads. g) Representative CFSE histogram of responder T cells cultured under each differentiation condition. h) Suppression of proliferation (%) was analyzed by flow cytometry. i) Treg stability was determined by assessing the remaining Foxp3^+^ cells among CD45.1^−^ CD4^+^ T cells after 3 days. All data are presented as mean ± S.D. Statistical significance was determined by Mann–Whitney test in b–e) and h–i) Wilcoxon test in f). n.s. = nonsignificant, *p < 0.05, **p < 0.01, ***p < 0.001.

We first generated a synthetic chimeric peptide comprising the cytoplasmic domain of CTLA‐4 and BBB‐permeable peptide dNP2 connected by a short linker (**Figure** **1**a). The peptide was purified using trifluoroacetic acid salt and had a purity above 95% and was stable at 25 °C (Figure S1, Supporting Information). To determine whether this synthetic CTLA‐4 signaling peptide was able to induce Foxp3^+^ Tregs in vitro, we stimulated sorted naïve T cells (CD4^+^CD25^−^CD62L^high^CD44^low^) with anti‐CD3 and anti‐CD28 antibodies (Th0, Figure 1b), or differentiated them to effector T cells including Th1 (Figure 1c), Th17 (Figure 1d), and iTreg (Figure 1e) cells with TGF‐*β*. dNP2‐ctCTLA‐4 peptide treatment resulted in a significant increase in expression of Foxp3 and CD25 under all conditions but decreased production of cytokines IFN‐*γ* and IL‐17 in a dose‐dependent manner (Figures S2 and S3, Supporting Information). The IC_50_ value was 1.652 × 10^−6^ m under Th17 conditions, and 2 × 10^−6^ m was used as the optimal dose in vitro in most of our data. The Foxp3^+^ T cells induced by dNP2‐ctCTLA‐4 peptide showed increased Treg markers, such as CD25 and CD73 compared to Foxp3^+^ T cells cultured under Th17 conditions, but the expression level of CTLA‐4 remained unchanged (Figure 1f). In addition, these Foxp3^+^ T cells significantly suppressed responder T cell proliferation, like iTregs, compared to Foxp3‐expressing cells cultured under Th17 conditions (Figure 1g,h). Interestingly, the expression of Foxp3 decreased rapidly in Th17 cells during responder activation and proliferation; in contrast, dNP2‐ctCTLA‐4‐induced Foxp3^+^ T cells and iTregs showed sustained Foxp3 expression (Figure 1i). These results collectively suggest that the CTLA‐4 signaling peptide can transmit signals to T cells to stably express Foxp3 and differentiate into regulatory T cells. Moreover, these Foxp3^+^ T cells have a comparable suppressive function to iTregs, even in the presence of inflammatory cytokine

### 2.2 The Lysine Motif in ctCTLA‐4 is Critical for Foxp3 Induction and EAE Inhibition

**Figure 2:**
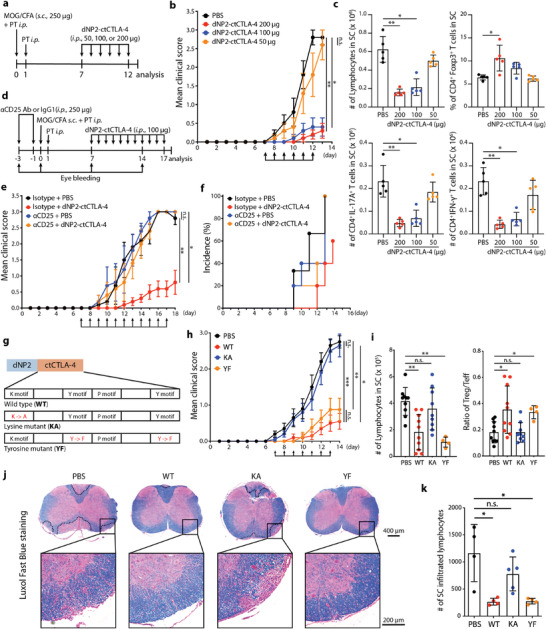
The lysine motif of CTLA‐4 is required for Foxp3 induction and Treg‐mediated EAE suppression. a–c) Experimental autoimmune encephalomyelitis (EAE) was induced by MOG_35–55_ in CFA emulsion with PTX. After immunization, C57BL/6 mice were treated with different doses of CTLA‐4 signaling peptide (50, 100, 200 µg, *i.p*.) from days 7 to 12 (5 mice per group, representative data from two independent experiments). a) Experimental scheme used to induce EAE. b) Clinical score of EAE. c) Lymphocytes that had infiltrated the spinal cord were analyzed by flow cytometry on day 13. d–f) EAE was induced in mice depleted of Tregs using *α*‐CD25 antibody (250 µg, two times *i.p*. injections). After immunization, C57BL/6 mice were treated with CTLA‐4 signaling peptide (100 µg, *i.p*.) every day from days 7 to 17 (5 mice per group, representative data from two independent experiments). d) Experimental scheme of Treg depletion in the EAE mouse model. e) Clinical score of Treg‐depleted EAE mice. f) Percent of EAE incidence in the Treg‐depleted EAE model. g–k) EAE was induced by MOG_35–55_ in the CFA emulsion with PTX. After immunization, mice were treated with CTLA‐4 WT, KA, or YF peptide (100 µg, *i.p*.) every day from days 7 to 13 (4–10 mice per group, two independent experiments). g) Schematic images of the WT synthetic CTLA‐4 signaling peptide (WT), the lysine mutant (KA), and the tyrosine mutant (YF). h) EAE clinical score. i) Number of infiltrated lymphocytes (left) and Treg/Teff ratio (right) in the spinal cord were analyzed at day 14. j) Histological analysis of spinal cord tissue stained with Luxol fast blue at day 14. The area of demyelination was marked with a dashed black line. k) Quantification of infiltrated lymphocytes in a section of spinal cord using ImageJ software. Data are presented as mean ± S.E.M. in b,e,h) and mean ± S.D. in c,i,k). Statistical significance was determined by Friedman test with post‐hoc Dunn's test in b,e,h) or Kruskal–Wallis test in c,i,k). n.s. = nonsignificant, *p < 0.05, **p < 0.01, ***p < 0.001.

To evaluate the potential utility of dNP2‐ctCTLA‐4 for treatment of diseases characterized by increased Treg function, we set up a semitherapeutic scheme of treatment in a mouse experimental autoimmune encephalomyelitis (EAE) model (**Figure** **2**a). dNP2‐ctCLTA‐4 peptide (50–200 µg) was administered every day from day 7 of immunization with MOG_35–55_ peptide antigen, and the clinical score decreased significantly with 100  and 200 µg peptide treatment (Figure 2b). The number of spinal cord‐infiltrating lymphocytes and IL‐17A‐ or IFN‐*γ*‐producing CD4 T cells decreased significantly in a dose‐dependent manner, while the proportion of Foxp3^+^ CD4 T cells increased (Figure 2c), suggesting that the inhibition of EAE disease progression by dNP2‐ctCTLA‐4 might be related to an in vivo increase in Tregs. dNP2 peptide alone has no effects on Foxp3 expression and EAE progression (Figure S4, Supporting Information). To further confirm Treg‐mediated regulation of autoimmune disease, we depleted endogenous CD25 positive regulatory T cells using anti‐CD25‐neutralizing antibodies 3 days and 1 day prior to MOG immunization (Figure 2d; and Figure S5, Supporting Information). dNP2‐ctCTLA‐4 did not improve EAE clinical score or disease incidence with Treg depletion, suggesting that the inhibition of EAE by dNP2‐ctCTLA‐4 is based on regulation of Treg function in vivo (Figure 2e,f).

Two tyrosine‐motifs in the cytoplasmic domain of CTLA‐4 recruit phosphatases like SHP or PP2A to regulate TCR signaling.^[^ ^7^ ^]^ A recent study demonstrated that the lysine motif is important for endocytosis of B7, which is expressed by antigen presenting cells, via binding to PKC‐*η*.^[^ ^8^ ^]^ Thus, we generated a lysine motif mutant (KA) and an all‐tyrosine‐motif mutant (YF) of the cytoplasmic domain of CTLA‐4, linked these with dNP2 (Figure 2g), and evaluated the functions of the mutant peptides in vitro and in an EAE mouse model. Under in vitro Th17 culture conditions, only the KA mutant did not induce Foxp3 (Figure S6, Supporting Information). Moreover, the ΚΑ mutant did not reduce clinical score, whereas both WT and the YF mutant showed comparable EAE disease regulation (Figure 2h). The number of spinal cord‐infiltrating cells was reduced by WT and YF peptide treatment and there was an increase in the population of Tregs, whereas the KA mutant had no effect on spinal cord‐infiltrating cells or the number of Tregs (Figure 2i). Spinal cord tissue histology also demonstrated reduced demyelination and tissue infiltration by inflammatory cells in mice treated with WT and YF peptide but not with KA peptide (Figure 2j,k). These results collectively suggest that dNP2‐ctCTLA‐4 peptide treatment has a significant therapeutic function in EAE disease progression, mediated by in vivo generation of Tregs and the lysine motif in ctCTLA‐4 is critically required for Foxp3 induction and inhibition of disease progression.

### 2.3 Transcriptomic and Proteomic Analysis of Foxp3‐Positive Cells Induced by dNP2‐ctCLTA‐4

**Figure 3:**
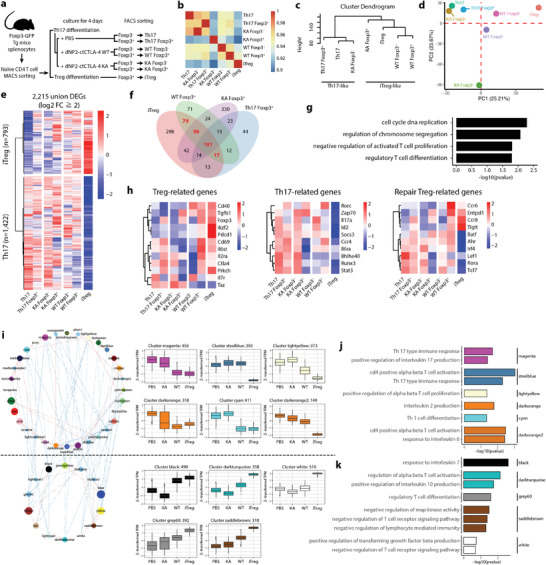
Transcriptome analysis of Foxp3 positive cells induced by dNP2‐ctCTLA‐4 showing the similarity of these cells to iTreg. a) Scheme of RNA‐seq sample preparation. Foxp3 was induced by 2 × 10^−6^ m of peptide under Th17 conditions. b–d) The distance between samples was visualized by b) a heatmap of the correlation coefficient between samples, c) a dendrogram based on the “average method,” and d) a PCA plot using 34 432 expressed transcripts. e) A heatmap of differentially expressed genes (DEGs, log_2_‐fold change ≥ 2 & FDR < 0.01) was drawn to compare Th17 and iTregs. f,g) dNP2‐ctCTLA‐4‐treated Foxp3^+^ Th17 cells showed an iTreg‐like transcription pattern. f) Venn diagram of upregulated genes versus Th17 and g) bar plot of significantly enriched (FDR < 0.2) GO terms for genes commonly upregulated in iTregs and WT Foxp3^+^ cells (log_2_ fold change ≥2 vs Th17). h) Comparison of expression of Treg‐related, Th17‐related, and repair Treg‐related genes among samples. i) Coexpression network of module eigengene vectors (left) and boxplot showing scale‐normalized (Z‐score) expression of modulized genes (blue line: correlation coefficient < −0.5; red line, correlation coefficient > 0.5; node color, color‐labeled modules; node size, number of genes in each module). j,k) Significantly enriched functions of upregulated modular genes in j) Th17 cells and k) iTregs.

Next, to further confirm the ability of dNP2‐ctCTLA‐4 to induce Tregs during Th1 or Th17 differentiation under inflammatory cytokine stimulation, we performed comparative transcriptomic and proteomic analyses of T cells (Foxp3 positive vs negative) stimulated with WT or KA mutant dNP2‐ctCTLA‐4 peptide (**Figure** **3**a). Correlation coefficient analysis revealed that Foxp3‐positive cells treated with WT peptide had the most transcriptomic similarity with iTregs, and even Foxp3‐negative cells treated with WT peptide showed closer similarity to iTregs than KA‐treated Foxp3‐positive cells or Th17 group cells (Figure 3b). Cluster dendrogram (Figure 3c) and the principal component analysis (PCA) plot (Figure 3d) also demonstrated that Foxp3^+^ T cells induced by dNP2‐ctCTLA‐4 WT under Th17 conditions were more like iTregs than dNP2‐ctCTLA‐4 KA‐treated cells or other controls. We identified a total of 2215 differentially expressed genes (DEGs) with over fourfold change between iTreg and Th17 samples. A heatmap of differentially expressed genes showed that the Foxp3^+^ population treated with dNP2‐ctCTLA‐4 WT had low Th17 gene expression and similar gene expression patterns to iTregs compared with KA mutant samples (Figure 3e). The Venn diagram of DEGs increased in iTregs showed that the Foxp3^+^ population treated with dNP2‐ctCTLA‐4 WT had more genes in common with iTregs than other Foxp3^+^ populations (Figure 3f). Gene ontologies of negative regulation of activated T cell proliferation and regulatory T cell differentiation were obtained (Figure 3g). We further analyzed the expression of well‐known signature genes of Tregs, Th17 cells, and repair Tregs (Figure 3h). The dNP2‐ctCTLA‐4 WT‐treated Foxp3^+^ population exhibited an iTreg‐like transcriptomic phenotype characterized by elevated expression of *Foxp3*, *Tgfb1*, *Il6st*, *Il2ra*, and *Ctla4* and low expression of *Il7r* and *Taz*. In particular, the expression level of *Taz*, a recently discovered critical coactivator of ROR*γ*t,^[^ ^11^ ^]^ was decreased only in iTreg and dNP2‐ctCTLA‐4 WT‐treated populations, whereas Foxp3^+^ Th17 and dNP2˜‐ctCTLA‐4 KA‐treated cells showed high expression of *Taz*. Interestingly, the dNP2‐ctCTLA‐4 WT‐treated Foxp3^+^ population had higher expression levels of *Ccr6*, *Entpd1*, *Batf*, *Ahr*, *Irf4*, and *Rora* than iTregs, similar to what has been reported for repair Tregs or ROR*γ*t^+^ T regulatory 17 (Tr17) cells.^[^ ^12^ ^]^

To further identify the potential biologic consequences of increased expression of specific genes, we clustered gene sets that were coexpressed and had similar expression patterns and constructed gene coexpression networks from transcript expression profiles for all samples, which are represented as modules. A total of 46 modules were identified and labeled with different colors (Figure S7, Supporting Information). Since these modules share similar biological functions, we analyzed each module through gene ontology (GO) and kyoto encyclopedia of genes and genomes (KEGG) pathway analyses. The intermodular network showed two distinguishable groups of modules, the upper and lower groups, representing sets of genes highly expressed by Th17 and Tregs, respectively. The box plot shows representative modules in each group (Figure 3i). Expression patterns within each module revealed that dNP2‐ctCTLA‐4 WT‐treated cells had expression profiles more similar to iTregs than dNP2‐ctCTLA‐4 KA cells, with significant enrichment of genes involved in regulatory T cell differentiation (Figure 3k). Modules that characterized the dNP2‐ctCTLA‐4 KA mutant group and Th17 cells were enriched in Th17‐type immune response genes in addition to genes involved in “GO: positive regulation of interleukin 17 production,” “GO: positive regulation of alpha beta T cell proliferation,” and “GO: response to interleukin 6” (Figure 3j; and Table S1, Supporting Information). In addition, RNA‐seq analysis of cells under Th1 differentiation conditions consistently revealed that dNP2‐ctCTLA‐4 peptide treatment increased Treg characteristics including *Il2ra, Entpd1, Ctla‐4, Icos, Havcr2, Il2rb*, and *gata1* with transcriptional similarity to iTregs (Figure S8, Supporting Information), collectively suggesting that dNP2‐ctCTLA‐4 switches the effector T cell fate from Th1 or Th17 cells to Treg‐like cells.

### 2.4 Proposed Functional Mechanism by Which dNP2‐ctCLTA‐4 Increases Foxp3 Expression

**Figure 4:**
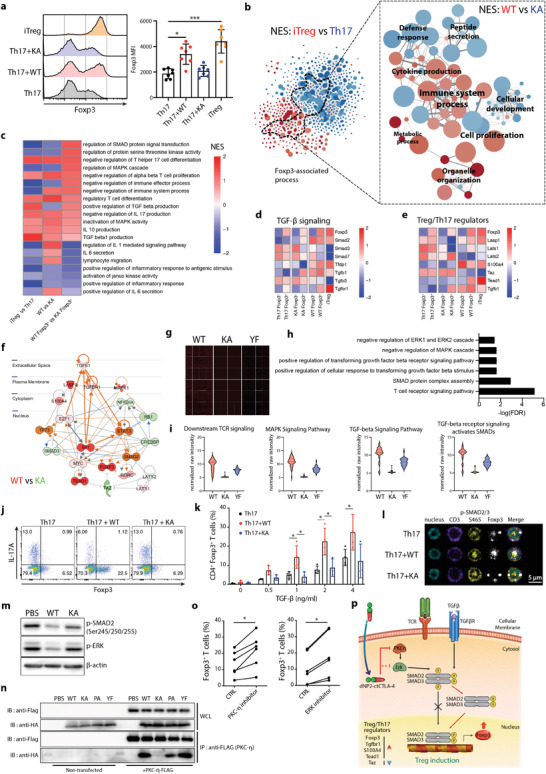
The lysine motif of CTLA‐4 is required for Foxp3 induction and increased sensitivity to TGF‐*β* signaling. a) Representative histogram of Foxp3 expression (left) and mean fluorescence intensity (right) in Foxp3^+^ populations (*n* = 7). b) GO network of DEGs between iTreg and Th17 (|log_2_ fold change| ≥ 2) (left) and Foxp3‐associated subnetworks based on normalized enrichment scores (NES) between WT and KA‐treated Th17 cells (both Foxp3^+^ and Foxp3^−^) (red, positive NES between WT and KA; blue, negative NES between WT and KA) (right). c) A heatmap of NES between groups labeled on the *x*‐axis. d,e) A heatmap of target genes involved in d) TGF‐*β* signaling and e) Treg/Th17 regulator. f) Predicted upstream regulatory network response to dNP2‐ctCTLA‐4 WT‐treated Foxp3^+^ cells based on IPA. g–i) Binding protein target analysis of the cytoplasmic domain of CTLA‐4 using Huprot proteome microarray data. g) Total chip images of each group. h) GO analysis of binding between CTLA‐4 WT/KA/PA/YF and human proteins (z‐score > 1, fold change > 2). i) Normalized raw intensity of selected GO terms. j,k) In vitro induction of Foxp3 under Th17 conditions dependent on TGF‐*β*. Mouse naïve CD4 T cells were stimulated with anti‐CD3/CD28 mAb in the presence or absence of 2 × 10^−6^ m WT or KA CTLA‐4 signaling peptide for 4 days (*n* = 4). j) Representative FACS dot plot and k) bar graph indicating TGF‐*β* dose‐dependent Foxp3 induction. l) Intranuclear localization of p‐Smad2/3 analyzed by confocal microscopy under Th17 differentiation conditions. m) Immunoblotting of the p‐Smad2 linker region. Peptide‐treated mouse splenocytes (2 × 10^−6^ m) were stimulated with anti‐CD3/28 and 5 ng mL^−1^ of TGF‐*β* for 30 min. n) Interaction between the lysine motif of CTLA‐4 and PKC‐*η* as assessed by immunoprecipitation and immunoblotting assay. HEK293 T cells were transfected with PKC‐*η*‐Flag‐expressing vector for 48 h. After 48 h, cells were treated with 5 × 10^−6^ m peptide for 2 h. o) Mouse naïve CD4 T cells were stimulated with anti‐CD3/CD28 mAb in the presence or absence of 5 × 10^−6^ m of PKC‐*η* pseudosubstrate inhibitor and 5 × 10^−6^ m of ERK inhibitor under Th17 differentiation conditions for 4 days. Foxp3 and IL‐17A expression was analyzed by flow cytometry (*n* = 6). p) Schematic image of the mechanism of TGF‐*β*‐dependent dNP2‐ctCTLA‐4‐mediated Treg induction. All data are presented as mean ± S.D. Statistical significance was determined by Kruskal–Wallis test in a,k) or Wilcoxon test in o) n.s. = nonsignificant, *p < 0.05, ***p < 0.001.

Notably, dNP2‐ctCTLA‐4 WT peptide treatment not only increased the proportion of Foxp3‐positive cells but also Foxp3 expression intensity compared to KA or Th17 group cells (**Figure** **4**a). As high intensity of Foxp3 expression has been shown to be correlated with suppressive activity in previous studies,^[^ ^13^ ^]^ we hypothesized that Foxp3 positive cells induced by dNP2‐ctCTLA‐4 WT would be more effective Tregs than cells treated with dNP2‐ctCTLA‐4 KA or Th17 cells. Network‐based gene set enrichment analysis (GSEA) was performed to verify differential expression of genes involved in Foxp3‐associated pathways. Subnetworks from differential GO functional networks were isolated and labeled by the normalized enrichment score (NES) in the dNP2‐ctCTLA‐4 WT versus KA group (both Foxp3^+^ and Foxp3^−^). Subnetwork analysis revealed differences between dNP2‐ctCTLA‐4 WT versus KA treatment, including differences in immune system processes, defense responses, cell proliferation, cytokine production, and cellular development (Figure 4b). In WT‐treated Foxp3 positive cells, NES patterns including negative regulation of IL‐17 production and Th17 differentiation were reduced, while SMAD signal transduction, mitogen‑activated protein kinase (MAPK) regulation, TGF‐*β*, and IL‐10 production were increased, suggesting that the lysine motif supports Treg differentiation over Th17 differentiation (Figure 4c). At the gene level, expression of TGF‐*β* signaling‐related genes like *Smad2*, *Tgfb1*, *Tgfbr1*, and *Foxp3* was increased by WT peptide treatment compared to KA peptide treatment (Figure 4d) and expression of Treg/Th17 regulators such as *Taz* and *Tead1* was similar to that observed in Tregs (Figure 4e). To clarify the signaling cascade from TGF‐*β* to Foxp3, we used Ingenuity Bioinformatics pathway analysis (IPA). The hypothesis we analyzed by IPA is that TGF‐*β* downstream signaling was enhanced by the lysine motif of dNP2‐ctCTLA‐4 peptide (Figure 4f).

In addition, we performed Huprot proteome microarray analysis by running peptides on protein‐coded chips to determine physical interactions between proteins (Figure 4g). We identified 5889 cytosolic proteins from among a total of 23 033 protein targets and analyzed functional GO terms (Figure S9, Supporting Information). Significantly interacting proteins were mostly related to MAPK signaling, SMAD, T cell receptor signaling, and transforming growth factor signaling (Figure 4h). We screened all functional motif mutants including KA and YF and found that the KA mutant exhibited loss of physical binding activity to target proteins (Figure 4i). These results collectively suggest that dNP2‐ctCTLA‐4 could control TGF‐*β* and MAPK signaling to induce Foxp3 and that the lysine motif is required for this.

To provide evidence for this hypothesis, naïve CD4 T cells were differentiated into Th17 cells with WT or KA mutant peptide with different amounts of TGF‐*β* added to the culture media. Increased Foxp3 expression was positively correlated with TGF‐*β* concentration in cells treated with WT peptide but not cells treated with KA mutant (Figure 4j,k); the same findings were obtained in Th1 cells (Figure S10, Supporting Information). SMAD2/3 activity is regulated by phosphorylation site. C‐terminal phosphorylation induced SMAD2/3 nuclear localization, leading to downstream gene expression of SMAD2/3, while phosphorylation of the linker region inhibited SMAD2/3 trans‐localization.^[^ ^14^ ^]^ Nuclear localization of C‐terminal phospho‐SMAD2/3 was significantly increased in WT‐treated cells compared to KA‐treated Th17 cells (Figure 4l). Because ERK is a negative regulator of SMAD activation via linker region phosphorylation,^[^ ^15^ ^]^ we determined the ERK activation and linker region phosphorylation of SMAD2. Phosphorylation of ERK and linker region phosphorylation of SMAD2 were significantly reduced by WT but not by KA peptide treatment (Figure 4m; and Figure S11a, Supporting Information), suggesting that TGF‐*β* signaling is increased by dNP2‐ctCTLA‐4 by regulation of ERK and translocalization of SMAD. PKC‐*η* has been suggested to interact with the lysine motif of CTLA‐4 and to be an important upstream activator of ERK.^[^ ^16^ ^]^ We therefore performed coimmunoprecipitation (co‐IP) experiments with HA‐tagged WT and mutant proteins and Flag‐tagged PKC‐*η*. There was complete loss of PKC‐*η* binding to ctCTLA‐4 in the KA mutant (Figure 4n; and Figure S11b, Supporting Information). In addition, both PKC‐*η* inhibitor and ERK inhibitor treatment during Th17 differentiation increased Foxp3 expression (Figure 4o). These results collectively suggest a hypothesis that dNP2‐ctCTLA‐4 induces Foxp3 expression during Th17 and Th1 differentiation via inhibition of the PKC‐*η*/ERK pathway, which increases TGF‐*β* signaling (Figure 4p).

### 2.5 dNP2‐ctCTLA‐4 Versus CTLA‐4‐Ig in Regulation of EAE

**Figure 5:**
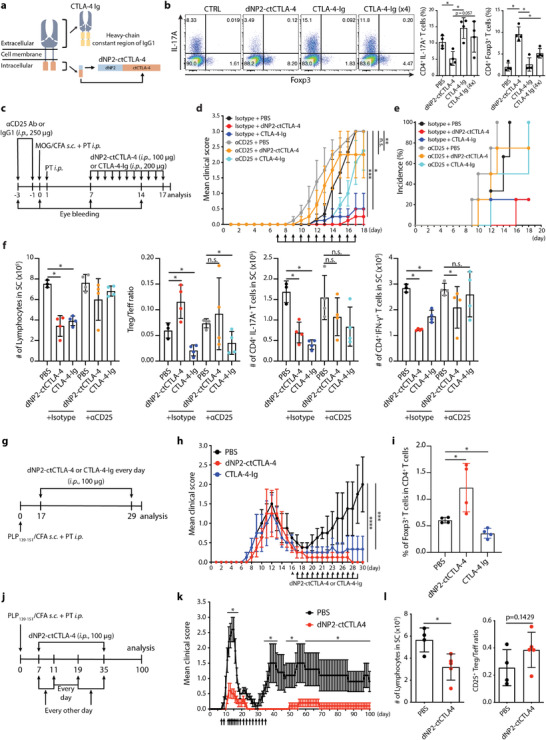
CTLA‐4 signaling peptide but not CTLA‐4‐Ig inhibits EAE progression via Treg induction and prevents relapse. a) Schematic images of CTLA‐4‐Ig and the synthetic CTLA‐4 signaling peptide. b) Representative dot plot (left) and bar graph (right) of Foxp3 induction. Mouse naïve CD4 T cells were cultured with irradiated splenocytes under Th17 differentiation conditions. Cells were treated with 5 × 10^−6^ m CTLA‐4 signaling peptide or 0.5 × 10^−6^ m or 2 × 10^−6^ m CTLA‐4‐Ig for 3 days (*n* = 4). c–f) Tregs were depleted by *α*‐CD25 antibody treatment (250 µg, injected 3 days and 1 day before immunization, *i.p*.) before EAE induction by MOG_35–55_ in CFA emulsion with PTX. After immunization, B6 mouse were treated with CTLA‐4 signaling peptide (100 µg, *i.p*.) or CTLA‐4‐Ig (200 µg, *i.p*.) from days 7 to 17 (4 mice per group). c) Experimental scheme showing establishment of a Treg‐depleted EAE mouse model treated with CTLA‐4 signaling peptide. d) Clinical score of Treg‐depleted EAE mice. e) EAE incidence of Treg depleted EAE model. f) Number of infiltrated lymphocytes (left), Treg/Teff ratio (middle), number of CD4^+^ IL‐17A^+^ T cells (middle), and CD4^+^ IFN‐*γ* ^+^ T cells (right) in the spinal cord were analyzed on day 18. g–i) EAE was induced in SJL mice by treatment with PLP_139–151_ in a CFA emulsion with PTX. After immunization, mice were treated with CTLA‐4 signaling peptideor CTLA‐4‐Ig (100 µg, *i.p*.) during the relapse phase, from day 17 to 29 (4 mice per group). g) Experimental scheme of the relapsing‐remitting EAE (SJL mouse) model. h) Clinical score of the relapsing‐remitting EAE model. i) Percent of Foxp3^+^ cells among CD4^+^ T cells in the spinal cord was analyzed on day 30. j–l) SJL was induced in mice by PLP_139–151_ in CFA emulsion with PTX. After immunization, mice were treated with CTLA‐4 signaling peptide (100 µg, *i.p*.) from day 7 (4–5 mice per group, representative data from two independent experiments). j) Experimental scheme of the relapsing‐remitting EAE mouse model. k) Clinical score of relapsing‐remitting EAE mice. l) Number of lymphocytes (left) and ratio of CD25^+^ Treg/Teff (right) in the spinal cord were analyzed on day 100. Data are presented as mean ± S.E.M. in d,h,k) and mean ± S.D. in b,f,i,l). Statistical significance was determined by Mann–Whitney test in b,f,i,k,l) or Friedman test with post‐hoc Dunn's test in d,h) n.s. = nonsignificant, *p < 0.05, **p < 0.01, ***p < 0.001, ****p < 0.0001.

CTLA‐4 function has been intensively highlighted with a focus on the extracellular domain of CTLA‐4 because of its ability to bind to B7, which is expressed by antigen presenting cells, competing with CD28.^[^ ^6a,b^ ^]^ A fusion protein of the extracellular domain of CTLA‐4 and the Fc region, named CTLA‐4‐Ig, was the first immune checkpoint regulatory drug approved for rheumatoid arthritis therapy.^[^ ^17^ ^]^ We hypothesized that CTLA‐4‐Ig would inhibit T cell activation but have no effect on Treg function, in contrast to the cytoplasmic domain of CTLA‐4 (**Figure** **5**a). We first utilized an in vitro coculture system of accessory APCs and sorted naïve CD4 T cells to differentiate cells into Th17 cells via treatment with dNP2‐ctCTLA‐4 or CTLA‐4‐Ig. At an equivalent dose of dNP2‐ctCTLA‐4 and CTLA‐4‐Ig, dNP2‐ctCTLA‐4 significantly inhibited IL‐17 production but increased Foxp3 expression (Figure 5b). There was a partial increase in Foxp3 expression in cells treated with a fourfold higher dose of CTLA‐4‐Ig than dNP2‐ctCTLA‐4, suggesting that Foxp3 expression is mainly induced by a signaling motif within CTLA‐4. Next, we performed in vivo comparative analysis of these molecules in an EAE mouse model after depleting Tregs prior to MOG_35–55_ immunization (Figure 5c). Without Treg depletion by anti‐CD25 antibody treatment, both dNP2‐ctCTLA‐4 and CTLA‐4‐Ig showed significant inhibition of EAE disease score and incidence; however, dNP2‐ctCTLA‐4 was ineffective after anti‐CD25 antibody treatment during the early and progression phases while CTLA‐4‐Ig was still able to control EAE disease at early phase (Figure 5d,e). The Treg/Teff ratio in the spinal cord was significantly increased by dNP2‐ctCTLA‐4 treatment but not by any of the other experimental or control treatments; CTLA‐4‐Ig treatment even reduced Treg numbers in vivo (Figure 5f). Due to the in vivo increase in the population of Tregs by dNP2‐ctCLTA‐4, we hypothesized that this peptide can be used to control disease relapse and potentially have utility in long‐term disease inhibition. The SJL mouse EAE model using the PLP_139–151_ peptide is more relevant to human multiple sclerosis as it has a relapse phase.^[^ ^18^ ^]^ Mice were treated with dNP2‐ctCTLA‐4 or CTLA‐4‐Ig on the day when disease relapse was observed (Figure 5g). Interestingly, both CTLA‐4 molecules were able to significantly inhibit disease relapse (Figure 5h). However, an increase in Foxp3^+^ CD4 T cells in spinal cord tissue was only observed in the dNP2‐ctCTLA‐4‐treated group, not the CTLA‐4‐Ig‐treated group (Figure 5i), suggesting differential functional mechanisms of these two molecules. Finally, we treated mice with dNP2‐ctCTLA‐4 during the first phase of disease onset and then stopped treatment to evaluate long‐term effects (Figure 5j). Even when we stopped treatment, mice treated with dNP2‐ctCTLA‐4 peptide exhibited sustained reductions in symptoms (Figure 5k) and number of spinal cord‐infiltrating lymphocytes (Figure 5l), as well as an increases in Treg/Teff ratio. These results collectively suggest that the cytoplasmic domain of the CTLA‐4 peptide can control T cell signaling to increase regulatory T cells in vivo, with potent and long‐term suppressive effects against EAE, thereby preventing disease relapse.

### 2.6 dNP2‐ctCTLA‐4 Peptide Treatment Increases the Population of Human Foxp3^+^ Tregs

**Figure 6:**
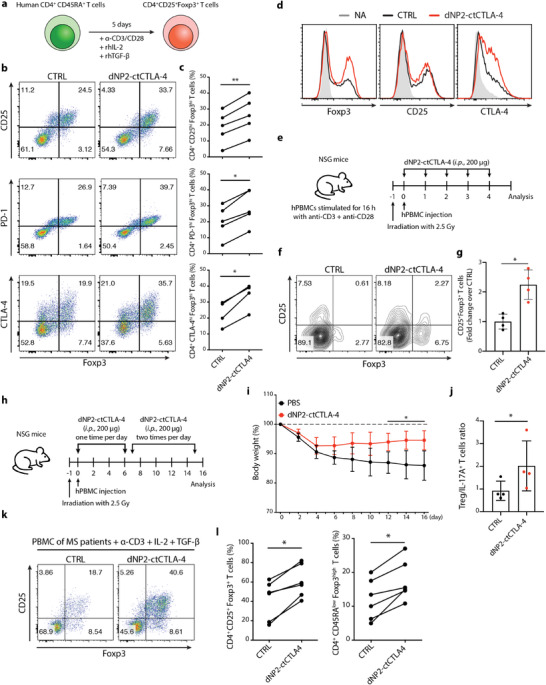
CTLA‐4 signaling peptide increases human Treg cells from healthy donors and MS patients. a–d) Human CD4^+^ CD45RA^+^ T cells were stimulated with human anti‐CD3/CD28 mAb under Treg differentiation conditions in the presence of 4 × 10^−6^ m CTLA‐4 signaling peptide for 5 days (*n* = 5). a) Experimental scheme of human naive T cell differentiation under Treg differentiation conditions. b) Representative flow cytometry data and c) line graph of CD4^+^ CD25^high^ Foxp3^high^ T cells, CD4^+^ PD‐1^high^ Foxp3^high^ T cells, and CD4^+^ CTLA‐4^high^ Foxp3^high^ T cells. d) Treg markers including Foxp3, CD25, and CTLA‐4 were expressed as a histogram to compare the Treg population proportions and Foxp3 staining intensity. e–g) In vivo human Foxp3^+^ Treg induction in NSG mice (4 mice per group, two independent experiments). e) Experimental scheme of the NSG mouse model. Human PBMCs were stimulated overnight and injected into irradiated NSG mice at day 0. The dNP2‐ctCTLA‐4 peptide (200 µg, *i.p*.) was injected into NSG mice on days 0–4. f) Representative flow cytometry data and g) bar graph of CD25^+^ Foxp3^+^ T cells isolated from the spleens of NSG mice on day 5. h–j) In vivo human Foxp3^+^ Treg induction in the GvHD model (4 mice per group, representative data from two independent experiments). h) Experimental scheme of the GvHD mouse model. Irradiated NSG mice were injected 24 h later with PBMC (2.5 × 10^6^ cells, *i.v*.). The CTLA‐4 signaling peptide (200–400 µg, *i.p*.) was injected into NSG mice from day 0 to 15. i) Body weight of the GvHD mouse model. j) Bar graph of Treg/IL‐17A cells ratio isolated from the spleens of NSG mice on day 16. k,l) PBMCs from MS patients were stimulated with human anti‐CD3 mAb under Treg differentiation conditions in the presence of 4 × 10^−6^ m CTLA‐4 signaling peptide for 3 days. CD4^+^ CD25^+^ Foxp3^+^ and CD4^+^ CD45RA^low^ Foxp3^high^ functional Tregs were analyzed by flow cytometry (*n* = 6). k) Representative flow cytometry data and l) line graph of CD25^+^ Foxp3^+^ T cells and CD45RA^low^ Foxp3^high^ T cells. Data are presented as mean ± S.E.M. in i) and mean ± S.D. in g,j). Statistical significance was determined by Wilcoxon test in c,l), or Mann–Whitney test in g,i,j). n.s. = nonsignificant, *p < 0.05, **p < 0.01.

We next sought to confirm whether dNP2‐ctCTLA‐4 can induce Foxp3 expression in human T cells. We utilized commercially available human naïve CD4 T cells to evaluate the effects of dNP2‐ctCTLA‐4 during activation of these cells with anti‐CD3 and CD28 antibodies and recombinant human IL‐2 and TGF‐*β* (**Figure** **6**a). CTLA‐4 signaling peptide treatment increased CD25^high^, PD‐1^high^, and CTLA‐4^high^ Foxp3^high^ T cell populations (Figure 6b,c). Numbers of Foxp3^+^, CD25^+^, and CTLA‐4^+^ cells were also increased after stimulation with CTLA‐4 signaling peptide (Figure 6d). Next, we transferred human peripheral blood mononuclear cells (PBMCs) to NSG mice after daily treatment of the mice with dNP2‐ctCTLA‐4 (Figure 6e) and harvested splenic human T cells. The CD25^+^Foxp3^+^ T cells were increased by dNP2‐ctCTLA‐4 treatment (Figure 6f,g), confirming the potential of this peptide to induce human Tregs in vivo. In addition, when human PBMCs were transferred into NSG mice and induced mild graft‐versus‐host disease (GvHD) (Figure 6h). dNP2‐ctCTLA‐4 treatment was able to reduce the loss in body weight (Figure 6i), increasing the ratio of Treg/IL‐17^+^ T cells (Figure 6j). Finally, we investigated whether CTLA‐4 signaling peptide could induce Foxp3 expression in multiple sclerosis (MS) patient PBMCs. PBMCs from MS patients treated with CTLA‐4 signaling peptide exhibited significantly increased CD25^+^ Foxp3^+^ regulatory T cells during their activation with TGF‐*β* and IL‐2 (Figure 6k,l; andTable S2, Supporting Information). In particular, there was significant elevation of CD45RA^low^Foxp3^high^ effector Treg cells (Figure 6l), collectively suggesting that dNP2‐ctCTLA‐4 could induce functional Treg cells both in healthy human T cells and MS patient T cells.

## 3 Discussion and Conclusion

In this study, we demonstrate a strategy for controlling autoimmune diseases by inducing Treg cells in vivo using a chimeric peptide of CTLA‐4. This CTLA‐4 signaling peptide (dNP2‐ctCTLA‐4) controls TGF‐*β* signaling to increase Foxp3 expression during Th1 or Th17 differentiation and expression of functional molecules like CD73, CD25, CTLA‐4, and PD‐1. In contrast to classical studies of CTLA‐4, which emphasized tyrosine motifs to regulate TcR signaling, we established that the lysine motif of ctCTLA‐4 is critical for Foxp3 expression and physical interaction with PKC‐*η*. We that proved its function is limited to ctCTLA‐4 and not CTLA‐4‐Ig for Treg cell induction, suggesting that this is a novel peptide for targeting Treg cells in vivo and controls autoimmunity.

MS is an autoimmune demyelinating disease with abnormal neuronal signaling in the central nervous system (CNS), resulting in a wide range of physical and mental symptoms.^[^ ^19^ ^]^ T cells in MS patients, especially Th1 and Th17 cells, have been shown to migrate into the CNS and to cause demyelination in the pathogenesis of MS.^[^ ^20^ ^]^ Current treatments have some limitations including risk of infection and lymphopenia (Fingolimod), development of progressive multifocal leukoencephalopathy, disease relapse (natalizumab), risk of secondary autoimmunity (alemtuzumab), or limited efficacy in highly active patients (first‐line disease‐modifying therapies).^[^ ^21^ ^]^ Although IL‐6, C4, and CD20 have recently been targeted due to their role in Th17 differentiation, complement‐based inflammation and B cells, respectively, with some success in specific patient populations, there are currently no drugs that allow long‐term immune regulation by direct targeting for Treg function.^[^ ^22^ ^]^ In addition, recent studies suggested that long‐term maintenance of immune regulation, rather than immune suppression, could be a new treatment paradigm in MS as a single treatment or in combination with current therapies.^[^ ^23^ ^]^ Loss of the suppressive function of CD4^+^CD25^+^ Tregs in MS patients with an increase in IFN‐*γ*‐producing Tregs was previously reported, and a negative correlation between Treg population and disease severity in MS patients has been reported, suggesting that immune homeostasis by Tregs is important in regulating MS.^[^ ^24^ ^]^ In addition, Treg transfer to an EAE animal model reduced disease severity with IL‐35 production and also contributed to myelin regeneration, suggesting that Tregs are actively involved in controlling and preventing CNS inflammation.^[^ ^25^ ^]^ In this study, the therapeutic effects of CTLA‐4 peptide were not limited to the initial phase of the disease, but also occurred in the relapse phase with long‐term regulation. Additionally, treatment of MS patient PBMCs with this peptide resulted in an increase the effector Tregs, implying its potential utility in the treatment of MS patients. CTLA‐4 peptide treatment also induced Treg cells in vivo in a psoriasis mouse model (Figure S12, Supporting Information). We thus speculate that the CTLA‐4 signaling peptide is a potential drug candidate for autoimmune diseases, such as MS and synergistic effects may be obtained in combination with other drugs.

Most regulatory T cells express Foxp3 to maintain tolerance to self‐antigens and prevent autoimmune disease.^[^ ^1a,b^ ^]^ CD25 is a major marker of suppressor cells and TGF‐*β* is an essential cytokine for Treg differentiation from naïve CD4 T cells.^[^ ^1c,d^ ^]^ A major mechanism by which suppressive Tregs function is paracrine IL‐10 production, prevention of the B7/CD28 interaction by CTLA‐4, and taking IL‐2 cytokine.^[^ ^1^, ^4^, ^5^ ^]^ Recently, therapeutic strategies of in vivo Treg induction for autoimmune diseases have been reported. The Bluestone group used an IL‐2 antibody complex with IL‐2 to increase Treg expansion in vivo and successfully inhibited type 1 diabetes development, GvHD, and EAE progression.^[^ ^26^ ^]^ In addition, the Sakaguchi group showed that by using a chemical compound “AS” to inhibit CDK9/18, they could increase differentiation of Tregs even from effector/memory T cells independently of TGF‐*β*, demonstrating prevention effects on EAE.^[^ ^27^ ^]^ More recently, Treg induction in MS patients by supplementation with short‐chain fatty acids resulted in a reduced annual relapse rate, indicating that increased Treg has a positive correlation with improved symptoms in MS patients.^[^ ^28^ ^]^ Here, we developed an alternative approach to increasing Treg cells in vivo for autoimmune disease therapy by utilizing the CTLA‐4 signaling peptide. Comprehensive RNA transcriptomic and proteomic analyses after dNP2‐ctCTLA‐4 peptide treatment of Th1 and Th17 cells revealed that induced Tregs expressed genes involved in tissue repair, such as *Entpd1*, *Batf*, *Irf4*, *Tigit*, and *Ahr*,^[^ ^12c–e^ ^]^ as well as Tr17‐related genes like *Ccr6*, and *Ccr8* that encode the proteins involved in infiltration of inflammatory sites.^[^ ^12b^ ^]^ Furthermore, CTLA‐4 signaling peptide‐treated Th17 cells showed increased expression of genes related to immune suppressive cytokines and cytokine receptors for Treg function, such as *Tgfb1*, *Il10*, *Il9*, *Il33*, *Il7r*, *Il27ra*, *Il2ra*, *Il2rb*, *Il6st*, and *Il1rl1* (Figure S13a, Supporting Information). IL‐10 and TGF‐*β* expression were also increased by the CTLA‐4 signaling peptide in the Th17 differentiation condition and spinal cord of the EAE model, indicating that these induced Tregs could have a suppressive function through IL‐10 and TGF‐*β* (Figure S13b,c, Supporting Information). Therefore, we speculate that this induction of Treg cells by dNP2‐ctCTLA‐4 infiltrated the CNS and efficiently controlled inflammation via IL‐10 and TGF‐*β*.

CTLA‐4, also known as CD152, is a CD28 homolog with higher avidity for B7 than CD28, and can negatively regulate costimulation of T cells, thereby functioning as an immune‐checkpoint supporting peripheral tolerance.^[^ ^6a^ ^]^ Abatacept (CTLA‐4‐Ig) is a fusion protein composed of the Fc region of immunoglobulin IgG1 and the extracellular domain of CTLA‐4.^[^ ^17a^ ^]^ Although abatacept has proven therapeutic efficacy in some autoimmune diseases, such as a rheumatoid arthritis, it failed clinical trials for other autoimmune diseases, including lupus nephritis and relapsing‐remitting MS (RRMS).^[^ ^29^ ^]^ The reason for the MS clinical failure of abatacept might be that the frequency of CD45RO^+^ memory Tregs was significantly reduced and the function of impaired Tregs could not be restored.^[^ ^24^, ^30^ ^]^ Interestingly, the number and function of Tregs reduced by abatacept recovered after treatment was discontinued.^[^ ^30^ ^]^ These observations could explain the limitations of long‐term immune regulation with abatacept alone in MS patients. In this study, we compared the modes of action of abatacept and dNP2‐ctCTLA‐4 and we demonstrated that dNP2‐ctCTLA‐4 increases the Treg population that hampers EAE development and progression, while abatacept treatment showed reduced Treg population compared to the control group correlated with previous report as descried.^[^ ^31^ ^]^ This result suggests obvious differences in the functions of the extracellular domain and intracellular domain of CTLA‐4 in T cell regulation. In a SJL mice model, the disease was still regulated for almost 100 days after discontinuation of dNP2‐ctCTLA‐4 treatment. This might be due to the presence of an increased number of Tregs to establish long‐term immune tolerance.

A previous study showed that a mutant form of CTLA‐4 unable to bind B7 ligand was still able to control cytokine production by T cells and type 1 diabetes development in mice.^[^ ^9d^ ^]^ Additionally, a ligand‐independent splicing variant lacking the extracellular domain of CTLA‐4 was able to inhibit T cell receptor signaling and cytokine production.^[^ ^9a–c^ ^]^ Thus, signaling modulation by the cytoplasmic domain of CTLA‐4 is constitutively activated and does not necessarily require its ligand interaction. In addition, we previously reported that the cytoplasmic domain of CTLA‐4 could control T cell activation and regulate inflammation in mice and humans.^[^ ^10a,c^ ^]^ The dNP2‐ctCTLA‐4 peptide could also inhibit TcR signaling like ZAP70 and ERK phosphorylation, which reduced IL‐2 production, while its lysine mutant did not exhibit the same inhibition (Figure S14, Supporting Information). There are several motifs of CTLA‐4, such as the lysine, tyrosine, and proline motif; tyrosine is an important residue that recruits SHP and regulates TcR signaling.^[^ ^7^, ^32^ ^]^ In this study, the tyrosine mutant of dNP2‐ctCTLA‐4 had a significant ability to induce Treg cells and regulate EAE disease, while the lysine mutant peptide completely lost the function of Treg induction and EAE prevention, suggesting that signal modulation by the lysine motif is critical for generation of Treg cells.

The molecular mechanism of Foxp3 induction by the CTLA‐4 signaling peptide appears to be molecular interaction with PKC‐*η* via its lysine motif; PKC‐*η* phosphorylates ERK, resulting in inhibition of SMAD activation and nuclear localization. Thus, the chimeric CTLA‐4 peptide described herein is dependent on TGF‐*β* signaling via inhibition of the PKC‐*η*/ERK pathway to increase Foxp3 expression. A recent study suggested that PKC‐*η* interaction to CTLA‐4 is required to reduce B7 expression in antigen‐presenting cells, which is an important mechanism of Treg function.^[^ ^8^ ^]^ However, our signaling peptide of CTLA‐4 is a different molecule that does not require binding to the B7 molecule. In fact, there was a significant increase of Foxp3 expression during Th1 and Th17 differentiation from naive CD4 T cells in PKC‐*η* KO mice (Figure S15, Supporting Information). However, there was no difference in EAE clinical score results between WT and PKC‐*η* KO mice (Figure S16, Supporting Information), which might imply an increase of Treg cells with loss of function in vivo. Therefore, PKC‐*η* binding to CTLA‐4 could be important in both Treg cell functions and Th1 and Th17 differentiation.

The ability of dNP2‐ctCTLA‐4 to induce Tregs in vivo and inhibit EAE development and relapse indicates that it could have curative potential against human autoimmune diseases, such as MS. However, this CTLA‐4 signaling peptide may have general limitations like other peptide drugs for clinical applications, such as a short half‐life. To overcome this, peptide optimization and modifications to increase in vivo stability, such as fatty acid conjugation and substitution of D‐amino acids, should be explored in preclinical studies. Before clinical trials can proceed, whether the Treg‐inducing approach is safe from serious side effects, such as tumor formation needs to be established. No serious side effects have been reported after treatment of MS with a propionic acid supplement in a phase II study that has been shown to increase Tregs,^[^ ^28^ ^]^ so we speculate that the dNP2‐ctCTLA‐4 peptide may also be relatively free from serious side effects. Further preclinical studies are needed to confirm that this candidate peptide drug is suitable for human clinical trials; the evidence presented here strongly indicates that increasing Tregs in vivo using this peptide drug may be a highly effective strategy for the treatment of autoimmune diseases.

## 4 Experimental Section

### Peptide Synthesis

Every peptide used in this paper, including dNP2‐ctCTLA‐4, mutant forms of dNP2‐ctCTLA‐4, and dNP2 was synthesized by solid‐phase peptide synthesis. Briefly, amino acids protected by Fmoc were sequentially conjugated to trityl chloride resin; then, the protecting groups of the resin and amino acid residues were removed through the global cleavage method to obtain crude under acidic conditions. The crude was purified by high performance liquid chromatography (Shimadzu, Kyoto, Japan) to obtain peptides, and finally freeze dried. The purity of the final peptide products was more than 95% (AnyGen, Kwangju, Korea).

### Cell Culture

HEK293T cells were purchased from the American Type Culture Collection and cultured in dulbecco's modified eagle's medium (DMEM, Cellgro; Mediatech, Washington, DC) supplemented with 10% fetal bovine serum (FBS, Welgene, Daegu, Korea) and 1% penicillin/streptomycin (Hyclone, Logan, UT). Cells were cultured at 37 °C in a 5% CO_2_ incubator (Forma Scientific, Marietta, OH).

### Mice

Six‐ to eight‐week‐old female C57BL/6 mice were purchased from Orient Bio (Daejeon, Korea), 8‐week‐old SJL mice were purchased from Charles River (Yokohama, Japan), and 10‐week‐old NSG mice were purchased from Joong Ah Bio (Suwon, Korea). Mice were housed and bred in a specific pathogen‐free animal facility at Hanyang University under controlled conditions with constant temperature (21 ± 1 °C), humidity (50 ± 5%), and a 12 h light/dark cycle with regular chow and autoclaved water. All mouse experimental procedures used in this study were approved by the Institutional Animal Care and Use Committees of Hanyang University. To reduce the number of animals used in vivo experiments based on the 3R principle, appropriate sample sizes (n) using G‐Power software version 3.1 were calculated. Briefly, it was analyzed with “ANOVA: Repeated measures, between factors” analysis using the following parameters: *α* err prob = 0.05, Power (1 – *β* err prob) = 0.8, and effect size from means. Mice studies were randomized in a blinded manner in EAE, GvHD, and psoriasis mouse models.

### Antibodies

Anti‐mouse CD4 (RM4‐5; 100 531; BioLegend), anti‐mouse CD25 (PC61; 102 016; BioLegend), anti‐mouse CD39 (Duha59; 143 809; BioLegend), anti‐mouse CD44 (IM7; 103 028; BioLegend), anti‐mouse CD62L (MEL‐14; 11‐0621‐82; eBioscience), anti‐mouse CD73 (TY/11.8; 127 223; BioLegend), anti‐human CD4 (RPA‐T4; 45‐0049‐42; eBioscience), anti‐human CD25 (BC96; 302 614; BioLegend), anti‐human CD45RA (HI100; 25‐0458‐42; eBioscience), and anti‐human PD‐1 (MIH4; 557 860; BD Bioscience) were used to stain the surface antigens of samples. For intracellular staining, anti‐mouse IFN‐*γ* (XMG1.2; 11‐7311‐81; eBioscience), anti‐mouse IL‐17A (eBio17B7; 12‐7177‐81; eBioscience), anti‐mouse CTLA‐4 (UC10‐4B9; 12‐1522‐81; eBioscience), anti‐mouse Foxp3 (FJK‐16s; 17‐5773‐82; eBioscience), anti‐human Foxp3 (259D; 320 208; BioLegend), and anti‐human CTLA‐4 (L3D10; 349 908; BioLegend) were used. For immunoblotting experiments, phospho‐Smad2 (Ser245/250/255) antibody (3104S; Cell Signaling Technology), phospho‐Smad2 (Ser465/467)/Smad3 (Ser423/425) antibody (D27F4; 8828S; Cell Signaling Technology), anti‐PKC‐*η* (PA5‐44 600; Invitrogen), anti‐phospho‐p44/42 MAPK (197G2; 4377s; Cell Signaling Technology), anti‐HA‐Tag (C29F4; 3724S; Cell Signaling Technology), anti‐FLAG (2368S; Cell Signaling Technology), goat anti‐rabbit IgG‐HRP (sc‐2030, Santa Cruz Biotechnology), and *β*‐actin (C4) (sc‐47 778, Santa Cruz Biotechnology) were used. For immunofluorescence, phospho‐Smad2 (Ser465/467)/Smad3 (Ser423/425) antibody (D27F4; 8828S, Cell Signaling Technology), anti‐mouse CD3e (145‐2C11; 12‐0031‐81; eBioscience), anti‐mouse Foxp3 (FJK‐16s; 17‐5773‐82; eBioscience), and anti‐rabbit IgG Alexa 488 (A11008, Invitrogen) were used.

### T Cell Differentiation

Naïve CD4 T cells were isolated from splenocytes of 6‐ to 8‐week‐old C57BL/6 mice using the mouse naïve CD4^+^ T Cell Isolation Kit (Miltenyi Biotec, Auburn, CA) according to the manufacturer's protocols. For RNAseq, naïve CD4 T cells from Foxp3‐GFP mice to sort Foxp3^+^ and Foxp3^−^ populations using a flow cytometry (FACS) Aria cell sorter III (BD Biosciences, Franklin Lakes, NJ) were purified. Purified naïve CD4 T cells were activated with plate‐bound anti‐CD3 (2 or 5 µg mL^−1^; 553 057; BD Pharmingen, La Jolla, CA) and anti‐CD28 (2 or 5 µg mL^−1^; 553 294; BD Pharmingen, La Jolla, CA) antibodies and differentiated with the following cytokine cocktails in a 96‐well plate for 3 or 4 days: media only for Th0; 5 µg mL^−1^ of anti‐IL‐4 (11B11; 16‐7041‐85; eBioscience), 50 U mL^−1^ of recombinant murine IL‐2 (212‐12; Peprotech, Rocky Hill, NJ), 2 ng mL^−1^ of recombinant murine IL‐12 (212‐12; Peprotech), 0.25 ng mL^−1^ of recombinant human TGF‐*β* (R&D Systems) for Th1; 5 µg mL^−1^ of anti‐IFN‐*γ* (XMG1.2; 16‐7311‐85; eBioscience), 5 µg mL^−1^ of anti‐IL‐4 (11B11; 16‐7041‐85; eBioscience), 2 ng mL^−1^ of recombinant murine TGF‐*β* (7666‐MB; R&D Systems, Minneapolis, MN), 30 ng mL^−1^ of IL‐6 (216‐16; Peprotech), 20 ng mL^−1^ of IL‐23 (1887‐ML‐101; R&D Systems), 20 ng mL^−1^ of IL‐1*β* (401‐ML‐010; R&D Systems), and 50 U mL^−1^ of IL‐2 (Peprotech) for Th17; 50 U mL^−1^ of IL‐2 (Peprotech) and 0.5 ng mL^−1^ of TGF‐*β* (R&D Systems) for Tregs. Cells were cotreated with peptides and cytokine cocktails. In some experiments, 5 × 10^−6^ m of PKC‐*η* pseudosubstrate inhibitor (sc‐3096; Santa Cruz) or 5 × 10^−6^ m of ERK inhibitor (UO126; 662005; Sigma) were also added.

### In Vitro Treg Assay

Naïve CD4 T cells from Foxp3‐GFP mice were isolated using a T cell isolation kit (Miltenyi Biotec). Sorted naïve CD4 T cells were cultured under Th17 differentiation conditions. At day 4, GFP^+^ cells were isolated using a FACS Aria III (BD Biosciences). Responder T cells were isolated using a CD4^+^CD45.1^+^CD25^−^CD44^low^CD62L^high^ marker from CD45.1^+^ cogenic mice by flow cytometry (FACS Aria III). Responder cells were labeled with 1.25 × 10^−6^ m CFSE (Invitrogen, Carlsbad, CA) for 10 min at room temperature and washed three times with PBS after which they were re‐suspended in RPMI 1640 medium supplemented with 10% FBS, 1% penicillin/streptomycin, 2 × 10^−3^ m l‐glutamine, 25 × 10^−3^ m HEPES, and 50 × 10^−6^ m *β*‐mercaptoethanol. Cells were stimulated with anti‐CD3/CD28 dynabeads (Invitrogen, Carlsbad, CA) for 3 days. GFP^+^ cells were cocultured with responder cells for an additional 3 days. T cell proliferation was assessed by flow cytometry.

### Flow Cytometry

Cells analyzed by flow cytometry were stained to exclude dead cells using the Zombie Aqua Fixable Viability Kit (Biolegend) before antibody staining at room temperature for 10 min. After washing, surface proteins of cultured naïve CD4 T cells were stained with monoclonal antibodies for 15 min at 4 °C. To determine intracellular cytokine levels, cells were restimulated with Cell Stimulation Cocktail (plus protein transport inhibitors) (ThermoFisher) for 4 h at 37 °C and stained with surface marker antibodies. Cells were then fixed, permeabilized using Foxp3/Transcription Factor Staining Buffer Set (eBioscience), and stained with anti‐mouse IFN‐*γ*, anti‐mouse IL‐17 and anti‐mouse Foxp3. Cells were analyzed using a FACS Canto II flow cytometer and FlowJo software version 10.7.

### EAE Induction

Eight‐week‐old female C57BL/6 mice were purchased from Orient Bio (Daejeon, Korea). The protocol described here was approved by the Animal Experimentation Ethics Committee of Hanyang University. EAE was induced by subcutaneous immunization using the MOG_35‐55_/CFA Emulsion PTX kit (Hooke Labs, Lawrence, MA) according to the manufacturer's protocol. Mice were anesthetized with isoflurane and MOG_35–55_/CFA was subcutaneously injected bilaterally, followed by pertussis toxin (PTX) intraperitoneal injection 2 and 24 h later. Mice were randomized to different groups after MOG immunization. EAE scores were assessed daily using the following scoring system: 0, no obvious signs of disease; 0.5, partially limp tail; 1, completely limp tail; 1.5, limp tail and waddling gait; 2, paralysis of one hind limb; 2.5, paralysis of one hind limb and partial paralysis of the other hind limb; 3, paralysis of both hind limbs; 3.5, ascending paralysis; 4, paralysis of trunk; 4.5, moribund; 5, dead.^[^ ^33^ ^]^ 50–200 µg of dNP2‐ctCTLA‐4 peptide or 100–200 µg of CTLA‐4‐Ig (Orencia, BMS) was injected intraperitoneally daily. Mice were euthanized at the end of the experiments; isolated spinal cords were digested with 1 mg mL^−1^‐ of DNase 1 (10 104 159 001; Sigma‐Aldrich) and 1 mg mL^−1^ of Collagenase D (11 088 866 001; Sigma‐Aldrich) at 37 °C and incubated at 80 RPM on shaker for 40 min. After enzyme digestion, 500 × 10^−3^ m ethylenediaminetetraacetic acid (EDTA) was added and lymphocytes were isolated by Percoll (GE Healthcare, Little Chalfont, UK) density‐gradient centrifugation. Isolated infiltrated lymphocytes were restimulated with Cell Stimulation Cocktail (plus protein transport inhibitors) (ThermoFisher) for 4 h at 37 °C and stained with anti‐mouse CD4. Cells were then fixed, permeabilized using the Foxp3/Transcription Factor Staining Buffer Set (eBioscience), and stained with anti‐mouse IFN‐*γ*, anti‐mouse IL‐17, or anti‐mouse Foxp3. Cells were analyzed using a FACS Canto flow cytometer and FlowJo software version 10.7.1. For histologic analysis, paraffin blocks of spinal cord tissues were deparaffinized and immersed in Luxol fast blue. For combination staining, hematoxylin and eosin were used (Dako). Infiltrated cells in the white matter region of spinal cord tissues were counted using Image J software version 2.0.0.

### Imiquimod‐Induced Psoriasis Model

The imiquimod‐induced psoriasis model was developed as described.^[^ ^34^ ^]^ Eight‐week‐old male C57BL/6 mice were obtained and 20 mg of Aldara cream (3 m Pharmaceuticals, Leicestershire, UK) was applied on the skin of each ear for 6 consecutive days under deep anesthesia with zoletil (30 mg kg^−1^) and rompun (10 mg kg^−1^) diluted in 180 µL PBS. Ear thickness was measured every day using a micrometer (Mitutoyo, Kawasaki, Japan). Then, 100 µg of dNP2‐ctCTLA‐4 peptide was injected intraperitoneally twice a day from day 0 to day 3 and three times a day from day 4 to day 5. On day 6, mice were sacrificed and CD4^+^ CD25^+^ Foxp3^+^ Tregs were analyzed in the spleen.

### Relapse‐Remitting Model

Eight‐week‐old female SJL mice were purchased from Charles River. EAE was induced by subcutaneous immunization with an PLP_139–151_/CFA Emulsion PTX kit (Hooke Labs, Lawrence, MA) according to the manufacturer's protocol. Mice were anesthetized with isoflurane and PLP_139–151_/CFA was subcutaneously injected at two sites on the upper back and lower back, followed by pertussis toxin (PTX) intraperitoneal injection immediately thereafter. Clinical scoring was performed as described above. 100 µm of dNP2‐ctCTLA‐4 peptide or 100 µg of CTLA‐4‐Ig (Orencia, BMS) was injected intraperitoneally daily or every other day during the early phase or relapse phase.

### In Vivo Treg Depletion

Anti‐CD25 antibodies (Bioxcell, 250 µg, *i.p*.) and isotype antibodies (Rat IgG1, *λ*, Bioxcell, 250 µg, *i.p*.) were injected at days −3 and −1 from the day of EAE induction. EAE was induced at day 0 using the MOG_35–55_/CFA Emulsion PTX kit (Hooke Labs, Lawrence, MA) as previously described above.

### In Vitro Coculture Assay

Naïve CD4 T cells and splenocytes from 2D2 mice were isolated. Splenocytes were irradiated with 3500 rad, then cocultured with naïve CD4 T cells in the presence of 20 ng mL^−1^ MOG peptide under Th17 differentiation conditions. For Th17 differentiation, 2 ng mL^−1^ of TGF‐*β*, 30 ng mL^−1^ of IL‐6, 5 µg mL^−1^ of *α*‐IFN‐*γ*, and 5 µg mL^−1^ of *α*‐IL‐4 were used. Cells were treated with 5 × 10^−6^ m of dNP2‐ctCTLA‐4 or 0.5 × 10^−6^ m or 2 × 10^−6^ m of CTLA‐4‐Ig for 3 days.

### RNA Sequencing

RNA was extracted from sorted Foxp3^+^ and Foxp3^−^ cells of PBS‐, dNP2‐ctCTLA‐4 WT‐, or dNP2‐ctCTLA‐4 KA‐treated T cells under Th17 conditions as well as iTregs with TRI Reagent RT (Molecular Research Center, Inc.) according to the manufacturer's protocol and quantified by UV spectrophotometry (CellTAGen, Inc., Seoul, Korea). Total RNA was used to construct cDNA libraries using the TruSeq Stranded mRNA LT Sample Prep Kit. The protocol consisted of polyA‐selected RNA extraction, RNA fragmentation, random hexamer‐primed reverse transcription, and 100 nt paired‐end sequencing using an Illumina NovaSeq 6000. Libraries were quantified using qPCR according to the qPCR Quantification Protocol Guide and quality was assessed using an Agilent Technologies 2100 Bioanalyzer. To estimate transcript expression, adaptor and low‐quality bases (minimum read length < 36 and < Q15) of all raw sequence reads were trimmed using Trimmomatic version 0.36. Clean reads were aligned to the mouse reference genome (ENSEMBL GRCm38) using STAR version 2.5.1a.^[^ ^35^ ^]^ Read counts and normalized expression values (TPM, transcripts per million) were estimated using RSEM version 1.2.31.^[^ ^36^ ^]^ Expressed genes were defined as those with  ≥ 5 read counts and ≥ 0.3 TPM in at least six samples. 34 432 of 142 647 expressed transcripts were used for further analysis. To identify DEGs and for functional analysis, read counts were normalized using edgeR^[^ ^37^ ^]^ in the TCC package of R Bioconductor. DEGs were defined at those with an absolute log_2_ fold change ≥ 2. To define the function of gene sets, the “clusterProfiler”^[^ ^38^ ^]^ package of R Bioconductor was used. A significantly enriched function was defined as an FDR < 0.2 using KEGG and GO databases from MsigDB (Broad Institute).^[^ ^39^ ^]^ To identify gene expression changes and functional associations, gene set enrichment analysis (GSEA, Broad Institute)^[^ ^39^, ^40^ ^]^ was conducted using the “GSEAPreranked” module. Gene ontology networks were constructed for significant GO biological processes (*p*‐value < 0.05). GO networks were represented using Cytoscape version 3.7. For weight gene–gene coexpression network analysis (WGCNA), pattern discovery was performed to identify dNP2‐ctCTLA‐4 treatment‐induced phenotypic changes from Th17 to iTregs. Log_2_‐transformed TPM values for all 34 432 expressed genes were used to estimate the similarity scores between genes and tree height was cut at 0.8. Total of 46 signed clusters were defined using WGCNA^[^ ^41^ ^]^ with a min cluster size of 100 (unclustered genes were classified into a gray module). Eigenvectors for each sample by cluster were calculated using the “moduleEigenegene” function of the WGCNA package in R. In transcription factor enrichment analysis for DEGs between dNP2‐ ctCTLA‐4 WT and KA mutant‐treated Th17 cells to determine upstream regulators induced by dNP2‐ctCTLA‐4 WT, enrichment scores for each transcription factor (TF) were calculated using “RcisTarget” of the SCENIC package in R and the reference mouse gene model (mm9‐ tss‐centered‐10kb).^[^ ^42^ ^]^ 220 DEGs (absolute log_2_ fold change ≥ 2 and *p*‐value < 0.005 for WT vs KA) were used to identify enriched TFs. Enriched TFs among up or downregulated DEGs after WT peptide treatment were defined to have a normalized enrichment score (NES) > 3.0 by default.

### Human Proteome Microarray

To identify ctCTLA‐4 binding partners, protein binding assays were performed with ctCTLA‐4 wild type (WT) and mutant form (KA, YF) peptides without a BBB‐permeable sequence using the Huprot Human proteome microarray version 3.1 (CDI lab). Microarray analysis was performed according to the manufacturer's protocols and arrays were scanned with a GenePix scanner. Signal intensities were acquired and analyzed using GenePix Pro (Molecular Devices, CA). Following the manufacturer's advice, signal intensity was defined as foreground median intensity (F635) to minimize signal bleeding in background regions around each spot. Data preprocessing was performed after two normalization steps. log_2_ transformation of all signal intensities before calculating z‐scores was performed. Z‐scores were expressed in terms of standard deviations from their means. Z‐scores have a distribution with a mean of 0 and a standard deviation of 1. The formula used to calculate the z‐score is shown below

(1) $$z=(I_{\mathrm{protein}}-I_{\mathrm{average}})/\mathrm{SD}$$

where *I*_protein_ is the fluorescence signal intensity of a spot, *I*_average_ is the mean fluorescence signal intensity of all protein spots, and SD is the standard deviation of all protein spots. A spot with a z‐score greater than 1.0 was considered a positive hit. Positive hits for each peptide sample were analyzed and visualized using R and Cytoscape ClueGO version 2.5.7.

### In Vitro Transfection

On day 0, 1 × 10^5^ HEK293 cells were seeded in DMEM containing 10% FBS and 1% penicillin/streptomycin and incubated for 24 h. Then, these cells were transfected with 1.5 µg FLAG‐tagged PKC‐*η* expressing vector using Lipofectamine 2000 (Invitrogen) on day 1. 48 h later, cells were further incubated with 5 × 10^−6^ m purified protein (dNP2‐ctCTLA‐4 WT, KA, PA, YF mutant) for 2 h. To eliminate cell surface binding proteins, cells were trypsinized and lysed with RIPA buffer containing 1 × 10^−3^ m NaF and 1 × 10^−3^ m phenylmethylsulfonyl fluoride (PMSF). dNP2‐ctCTLA‐4 and its mutant forms used in immunoprecipitation were purified using the *Escherichia coli* bacterial system as described previously.^[^ ^10a^ ^]^

### Immunoprecipitation

After transfection, lysates were incubated with anti‐FLAG (A2220; Sigma) antibodies covalently attached to agarose for 2 h at 4 °C on a rotator. Beads were then collected by centrifugation at 300 *g* for 1 min at 4 °C and washed with 1 mL cold cell lysis buffer (50 × 10^−3^ m Tris‐HCl pH 7.4, 1 × 10^−3^ m EDTA, 1 × 10^−3^ m EGTA, 1 × 10^−3^ m NA_3_VO_4_, 10 × 10^−3^ m *β*‐glycerophosphate, 50 × 10^−3^ m NaF, 5 × 10^−3^ m pyrophosphate, 270 × 10^−6^ m sucrose, and 1% Triton X‐100) five times. Finally, beads were resuspended in RIPA buffer containing 1 × 10^−3^ m NaF and 1 × 10^−3^ m PMSF and denatured with sodium dodecyl sulfate (SDS) sample buffer.

### Immunoblot Analysis

After T cell differentiation, cells were washed with PBS and lysed with RIPA buffer (Cell Signaling, Beverly, MA) containing 1 × 10^−3^ m NaF, 1 × 10^−3^ m PMSF, and Halt protease and phosphatase inhibitor cocktail (Thermo Fisher Scientific, Waltham, MA) for 30 min on ice. Immunoblotting was performed on PVDF membranes (Bio‐Rad) using the following primary antibodies: phospho‐Smad2, phospho‐Stat3, anti‐HA tag, anti‐Flag tag, and *β*‐actin mouse mAb.

### Immunofluorescence

After T cell differentiation, cells were washed with PBS and seeded on silane‐coated slide glass (5116‐20F, Muto) on a 12‐mm area marked using an ImmEdge Hydrophobic Barrier PAP pen (H‐4000, Vector). Cells on the slide glass were incubated for 1 h in a 37 °C incubator. After incubation, slides were washed twice with PBS to eliminate unattached cells. Then, attached cells were fixed with 4% paraformaldehyde (Wako Pure Chemical, Osaka, Japan) for 10 min at room temperature in the dark and permeabilized with DPBS supplemented with 1% BSA and 0.25% Triton X‐100 for 10 min at room temperature in the dark. After permeabilization, slides were blocked for 1 h with DPBS containing 1% BSA. Staining for SMAD2 and Foxp3 proteins was performed overnight on 4 °C followed by staining with anti‐rabbit IgG‐Alexa 488 and anti‐mouse CD3 antibodies for 2 h at room temperature in the dark. Nuclei were stained with Hoechst for 20 min at room temperature in the dark. Finally, cells were mounted with Dako Fluorescence Mounting Medium (S3023; Sigma) and visualized by confocal microscopy (Nikon).

### In Vitro Human Treg Induction

Human CD4^+^ CD45RA^+^ naïve CD4 T cells were purchased from LONZA (4W‐202). Human naïve CD4 T cells analyzed in this study were obtained from five healthy donors: four males (27, 43, 55, and 26 years old), one female (40 years old). Naïve CD4 T cells were stimulated with anti‐CD3 5 µg mL^−1^ (OKT3; 16‐0037‐85; ThermoFisher), anti‐CD28 5 µg mL^−1^ (CD28.2; 16‐0289‐85; ThermoFisher) in the presence of rhIL‐2 50 U mL^−1^ (200‐02; Peprotech) and rhTGF‐*β* (240‐b; R&D Systems) 5 ng mL^−1^ for 5 days. Cells were treated with 4 × 10^−6^ m dNP2‐ctCTLA‐4 WT peptide. On day 5, cells were stained with 1:1000 dilution of Live/Dead stain (Biolegend) for 10 min at room temperature, then stained with anti‐human CD4, anti‐human CD45RA, anti‐human CD25, or anti‐human PD‐1 for 30 min at 4 °C. Fixation and permeabilization (eBioscience) were performed for 40 min at room temperature followed by intracellular staining using anti‐human Foxp3 and antihuman CTLA‐4. Stained cells were analyzed using a FACS Canto II flow cytometer.

### In Vivo Human Treg Induction in NSG Mice

Human PBMCs were purchased from LONZA (4W‐270C). PBMCs analyzed in this study were obtained from two healthy donors; PBMCs were stimulated overnight with 5 µg mL^−1^ anti‐CD3 (ThermoFisher) and 5 µg mL^−1^ anti‐CD28 (ThermoFisher) mAb. Recipient 12‐week‐old NSG mice were irradiated with 2.5 Gy 24 h before PBMC injection. After overnight stimulation, PBMCs were washed with PBS and 6 × 10^6^ cells were resuspended in 200 µL PBS. PBMCs (200 µL) were injected intravenously into NSG mice. 200 µm of dNP2‐ctCTLA‐4 was injected intraperitoneally on days 0–4. On day 5, splenocytes were isolated and analyzed using a FACS Canto II flow cytometer. Studies of human naive T cells and human PBMCs were exempted by the Institutional Review Board of Hanyang University.

### GvHD in NSG Mice

Human PBMCs (LONZA, 4W‐270C) were washed with PBS and 2.5 × 10^6^ cells were resuspended in 200 µL of PBS. Recipient 12‐week‐old NSG mice were irradiated with 2.5 Gy 24 h before PBMC injection (*i.v*.), injected with 200 µL of human PBMCs and dNP2‐ctCTLA‐4 every day from day 0 to 15 (*i.p*.). Body weight was measured daily. On day 16, splenocytes were isolated and analyzed using a FACS Canto II flow cytometer.

### In Vitro Treg Induction of MS Patient PBMCs

Fresh PBMCs were isolated from the blood samples of three patients with MS^[^ ^43^ ^]^ using SepMate tubes (StemCell, #15450). All patients visited the MS/NMOSD clinic at Seoul National University Hospital, Republic of Korea between July 2020 and September 2020 and provided written informed consent prior to participation. MS patient PBMCs analyzed in this study were obtained from six donors: females 26, 32, 33, and 39 years old and males 31 and 35 years old. Demographic information of the MS patients is described in Table S2 (Supporting Information). This study was approved by the Institutional Review Board of Seoul National University Hospital (IRB number: H‐1902‐083‐1010). Fresh PBMCs were stimulated with 5 µg mL^−1^ anti‐CD3 mAb and incubated with 50 U mL^−1^ rhIL‐2 (Peprotech) and 2.5 ng mL^−1^ rhTGF‐*β* (R&D). They were treated with 4 × 10^−6^ m of dNP2‐ctCTLA‐4 peptide for 3 days. After 3 days, the number of CD4^+^CD25^+^Foxp3^+^ functional Tregs was analyzed by flow cytometry using a FACS Canto II.

### Enzyme‐Linked Immunosorbent Assay (ELISA)

Samples were collected from Th17 or iTreg differentiation conditions. All procedures for the IL‐10 ELISA (88‐7105; Thermo Fisher) and IL‐2 ELISA (431 004; Biolegend) were performed according to the manufacturer's instructions. Briefly, microwell plates (Corning Costar; 9018) were coated with capture antibodies overnight at 4 °C and blocked with ELISA/ELISPOT diluent 1X for 1 h at room temperature. Samples and twofold serial diluted IL‐10 standards were incubated at room temperature for 3 h, then detection antibodies and Streptavidin‐HRP were sequentially loaded. Between all procedures, plates were washed at least 3 times with wash buffer (1X PBS, 0.05% Tween‐20). 1X TMB solution was loaded and a stop solution (2 N H_2_SO_4_) was loaded. For analysis, the optical density was analyzed at 450 nm.

### Quantitative PCR

RNA from the spinal cord was extracted with RNAiso Plus (TaKaRa, Japan) according to the manufacturer's protocol and quantified by UV spectrophotometer (CellTAGen, Inc., Seoul, Korea). The cDNA was synthesized using the ReverTra Ace qPCR RT Master Mix (Toyobo, Osaka, Japan). Quantitative RT‐PCR was performed with iQ SYBR green supermix (Bio‐Rad, Hercules, CA) using a Bio‐Rad CFX Connect real‐time PCR detection system. Expression was normalized to *β*2m, as indicated in the figure legends.

### Statistical Analysis

All data were analyzed by nonparametric analysis using Mann–Whitney test, Kruskal–Wallis test, Wilcoxon test, or Friedman test with post‐hoc Dunn's test in GraphPad Prism version 7.0 (Graphpad Software, San Diego, CA). No data or outliers were excluded. Data are presented as mean ± S.D. or ± S.E.M. In all cases, significance was defined as *p* ≤ 0.05. Sample size and statistical analysis information is provided in each figure legend.

## Conflict of Interest

The authors have submitted a patent regarding peptides which induces Treg cells for autoimmune disease therapy. The patent is still pending approval.

## Author Contributions

G.‐R.K., W.‐J.K., and S.L. contributed equally to this work. J.‐M.C. conceived and supervised the study. G.‐R.K. designed and performed experiments including in vitro T cell differentiation, Treg suppression assays, EAE model analysis, and human sample experiments and W.‐J.K. designed and analyzed RNA sequencing, protein array, immunoblot, immunofluorescence data, quantitative PCR, and the psoriasis model. S.L. initiated and performed experiments including in vitro T cell differentiation, EAE model analysis, and immunoprecipitation. H.‐G.L. and J.‐H.K. supported the CTLA‐4‐Ig and immunoblot experiments. K.‐H.N. supported EAE model analysis. S.‐M.K. provided human MS patient samples. S.‐D.P. provided intellectual information on the project. J.‐M.C., G.‐R.K., and W.‐J.K. wrote the manuscript. All authors revised and approved the manuscript.

## Supporting information

Supporting Information

## Data Availability

The data that support the findings of this study are available from the corresponding author upon reasonable request.
