# Supplementary material for: In Vivo Induction of Regulatory T Cells Via CTLA‐4 Signaling Peptide to Control Autoimmune Encephalomyelitis and Prevent Disease Relapse
Source: Adv Sci (Weinh). 2021 May 5;8(14):2004973. doi: 10.1002/advs.202004973 (PMC8292875; doi:10.1002/advs.202004973)
Supplement: Supplementary file 1 — Supporting Information [file ADVS-8-2004973-s001.pdf]

© 2021 Wiley-VCH GmbH

ADVANCED SCIENCE Open Access

Supporting Information

for *Adv. Sci.*, DOI: 10.1002/advs.202004973

In vivo induction of regulatory T cells via CTLA-4 signaling peptide to control autoimmune encephalomyelitis and prevent disease relapse

Gil-Ran Kim, Won-Ju Kim, Sangho Lim, Hong-Gyun Lee,
Ja-Hyun Koo, Kyung-Ho Nam, Sung-Min Kim, Sung-Dong Park,
*and Je-Min Choi**

Supporting Information

In vivo induction of regulatory T cells via CTLA-4 signaling peptide to control autoimmune encephalomyelitis and prevent disease relapse

*Gil-Ran Kim, Won-Ju Kim, Sangho Lim, Hong-Gyun Lee, Ja-Hyun Koo, Kyung-Ho Nam, Sung-Min Kim, Sung-Dong Park, and Je-Min Choi**

a

Purity	Solubility	Molecular weight	Salt
97.2%	10 mg/ml	7.1 kDa	TFA

b

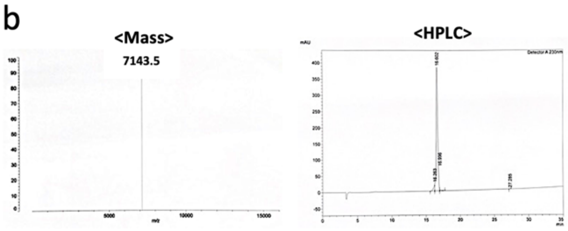

c

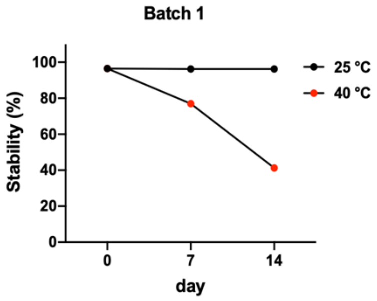

Figure S1. Physicochemical properties of synthetic dNP2-ctCTLA-4 peptide as determined by mass spectrometry and HPLC analysis.

a) Characteristics of the dNP2-ctCTLA-4 peptide. b) Molecular weight and purity of synthetic peptide were analyzed by mass spectrometry and HPLC, respectively. c) Stability of the dNP2-ctCTLA-4 peptide at 25°C or 40°C was analyzed by AnyGen Co., Ltd. Data are representative of two independent experiments. The peptide was incubated at 25°C or 40°C for 14 days and purity was assessed by HPLC.

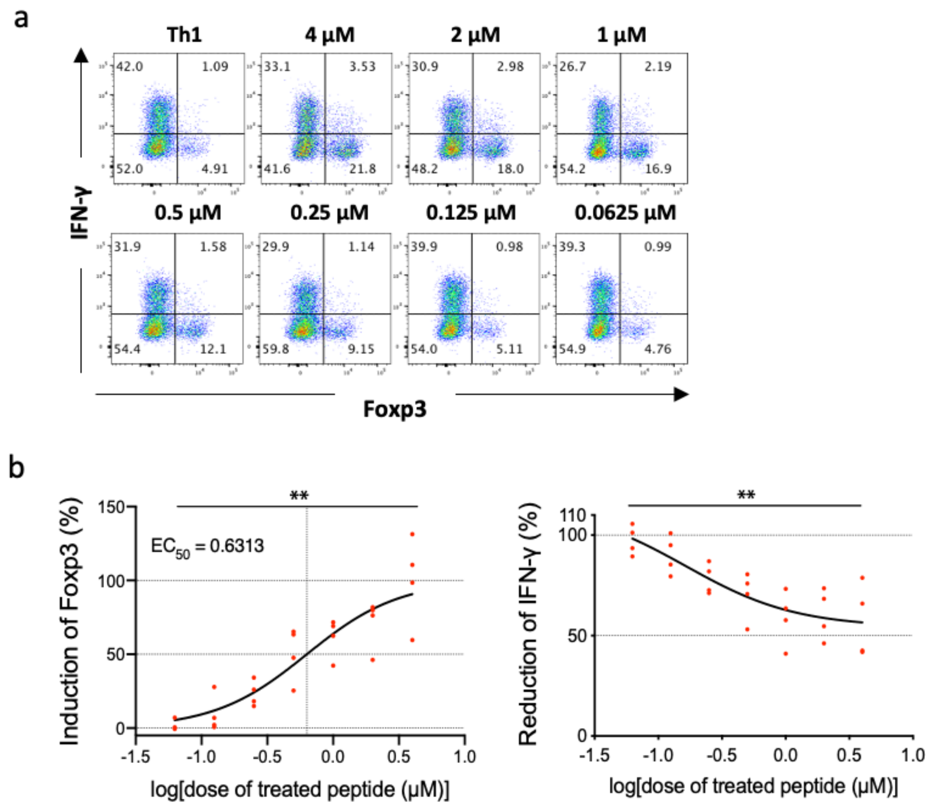

Figure S2. dNP2-ctCTLA-4 peptide inhibits Th1 differentiation and induces Foxp3 in a dose-dependent manner.

a) Dose-dependent Foxp3 induction and IFN-γ suppression by dNP2-ctCTLA-4 treatment under Th1 differentiation conditions. b) The EC_{50} value for Foxp3 induction and the IC_{50} for IFN-γ reduction under Th1 differentiation conditions (n=4). Each EC_{50} and IC_{50} value was calculated by nonlinear regression in XY analyses by Prism software. Statistical significance was determined by Kruskal-Wallis test in b. ***P*<0.01

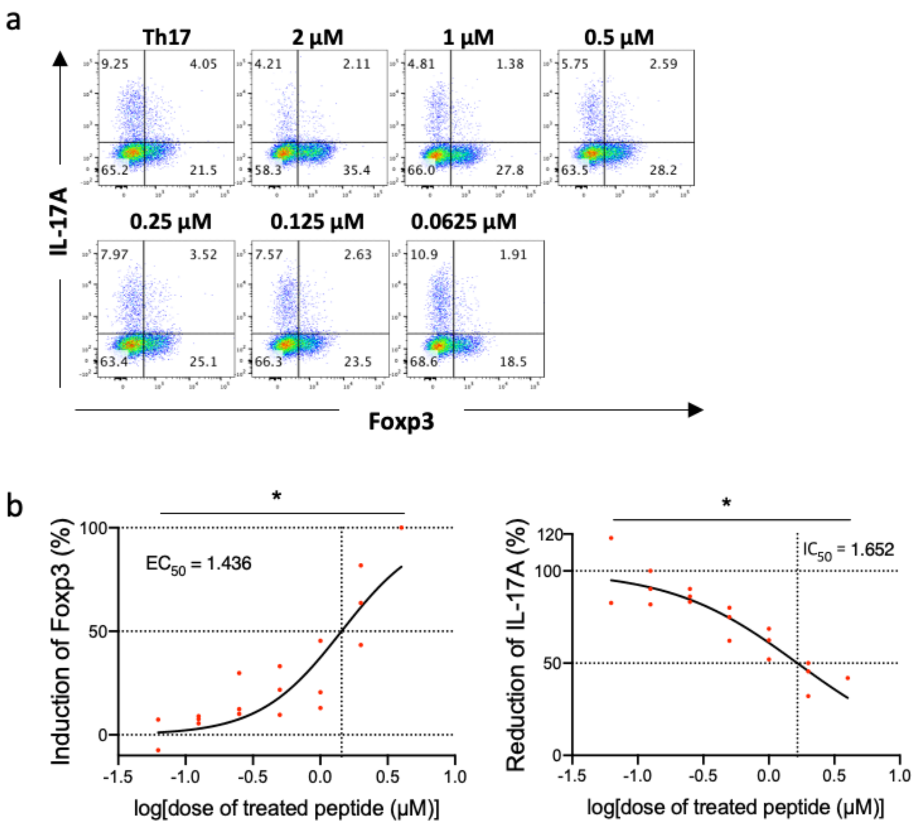

Figure S3. dNP2-ctCTLA-4 peptide inhibits Th17 differentiation and induces Foxp3 in a dose-dependent manner.

a) Dose-dependent Foxp3 induction and IL-17A suppression by dNP2-ctCTLA-4 treatment under Th17 differentiation conditions. b) EC_{50} of Foxp3 induction and IC_{50} of IL-17A reduction under Th17 differentiation conditions (n=1-3). Each EC_{50} and IC_{50} value was calculated by nonlinear regression in XY analyses by Prism software. Statistical significance was determined by Kruskal-Wallis test in b. $^*P<0.05$

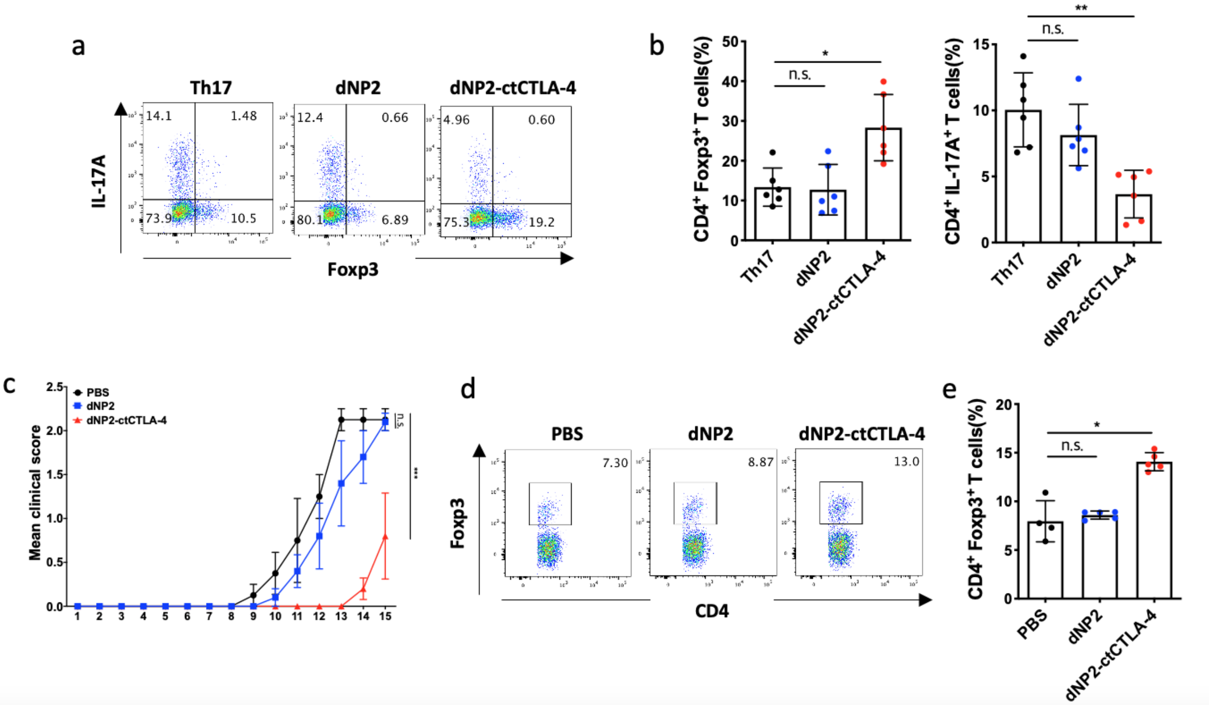

Figure S4. dNP2 alone could not inhibit Th17 differentiation and EAE progression.

a-b) Mouse naïve CD4 T cells were differentiated to Th17 cells in the presence of 2 µM of dNP2-ctCTLA-4 or dNP2 peptide (n=6). a) Representative dot plot and b) bar graph based on flow cytometry results obtained on day 4. c-e) Experimental autoimmune encephalomyelitis (EAE) was induced by MOG_{35-55} in CFA emulsion with PTX. After immunization, C57BL/6 mice were treated with dNP2-CTLA-4 or dNP2 peptide (100 µg, *i.p.*) from days 7 to 14 (4-5 mice per group, representative data from two independent experiments). c) Clinical score of EAE. d) Representative dot plot and e) bar graph of $CD4^+$ $Foxp3^+$ T cells in spleens. Data are presented as mean ± S.D. in b, e or mean ± S.E.M. in c. Statistical significance was determined by Kruskal-Wallis test in b, e or Friedman test with post-hoc Dunn's test in c. n.s. = non-significant, *P<0.05, **P<0.01, ***P<0.001.

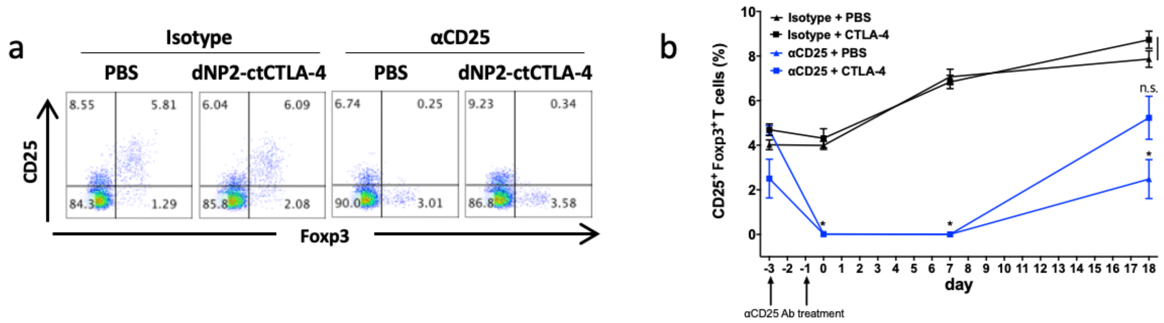

Figure S5. α-CD25 antibody treatment in mice depletes CD25^{+} Foxp3^{+} Tregs during EAE disease development.

a) Blood obtained from Treg-depleted mice was analyzed by flow cytometry on day 0 (5 mice per group). b) The percent CD4^{+} CD25^{+} Foxp3^{+} T cells in Treg-depleted mice was assessed on days -3, 0, 7, and 18. Data are presented as mean ± S.E.M. Statistical significance was determined by Kruskal-Wallis test in b. n.s. = non-significant, *P<0.05.

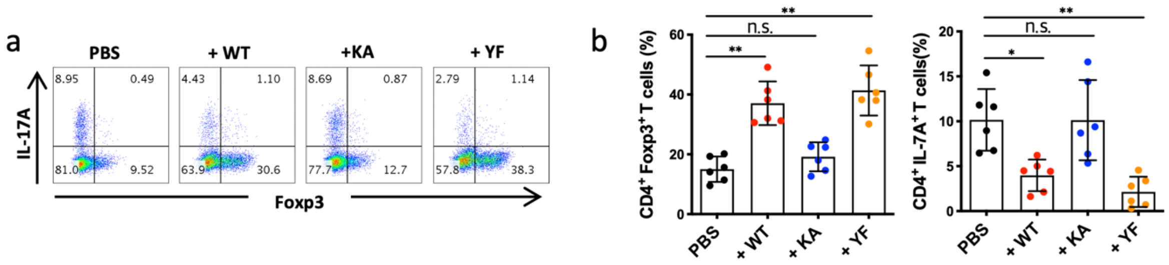

Figure S6. The lysine motif of ctCTLA-4 is required for Foxp3^{+} Treg induction *in vitro*. Mouse naïve CD4 T cells were differentiated to Th17 cells in the presence of WT or mutant peptides (n=6). a) Representative dot plot and b) bar graph based on flow cytometry results obtained on day 4. Data are presented as mean ± S.D. in b. Statistical significance was determined by Kruskal-Wallis test in b. n.s. = non-significant, *P<0.05, **P<0.01

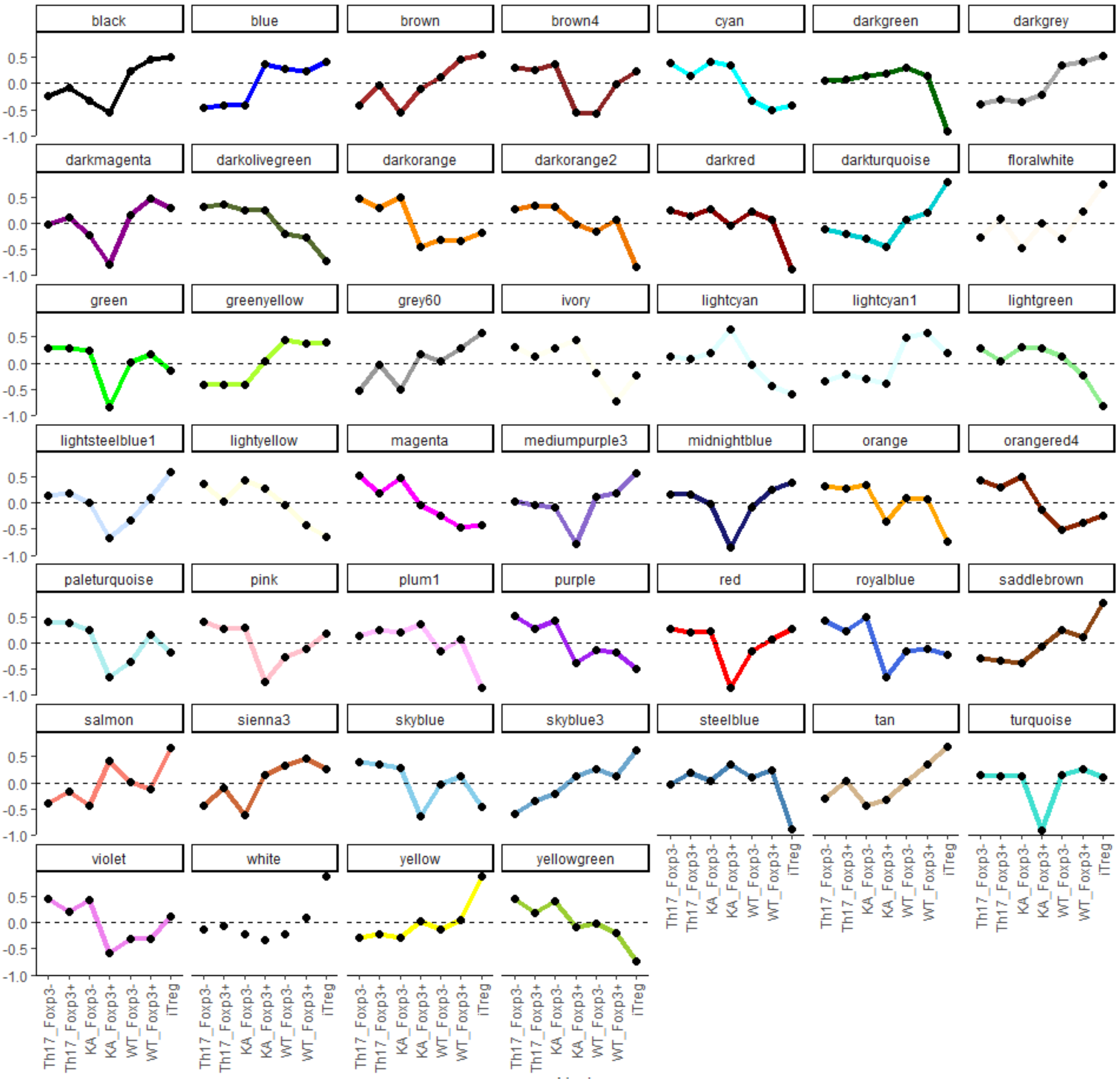

Figure S7. Forty-six modules were identified by eigengene vectors and indicate that the WT Foxp3^{+} group has an iTreg-like cluster pattern.

A total of 46 modules were identified by co-expression network analysis; expression of transcripts in each module is presented as a line plot.

Table S1. Functional enrichment analysis of each module indicating module characteristics of Th17 and iTregs.

GO terms with p-values less than 0.05 in each cluster were analyzed. GO terms, gene ratios, *p*-values, and gene IDs from each module in Figure 3I were analyzed.

cluster	Description	GeneRatio	BgRatio	pvalue	geneID
cyan	Th 1 cell differentiation	2/305	18/15838	0.046169	Lef1/Relb
darkorange	interleukin 2 production	4/247	63/15838	0.016772	Cd4/Ptprc/Sptbn1/Stat5b
	cd4 positive alpha beta T cell activation	4/113	86/15838	0.003329	Bcl3/Malt1/Sash3/Stat3
	response to interleukin 6	3/113	44/15838	0.003786	Sbno2/Socs3/Stat3
lightyellow	positive regulation of alpha beta T cell proliferation	2/283	19/15838	0.044549	Rasal3/Syk
magenta	Th 17 type immune response	3/344	27/15838	0.020202	Ly9/Prkcq/Stat3
	positive regulation of interleukin 17 production	2/344	15/15838	0.04099	Ly9/Prkcq
steelblue	CD4 positive alpha beta T cell activation	6/207	86/15838	0.000915	Batf/Cd86/Il4ra/Rorc/Satb1/Zc3h12a
	Th 17 type immune response	3/207	27/15838	0.005108	Batf/Rorc/Zc3h12a
black	response to interleukin 7	5/420	36/15838	0.002451	Atic/Atp5b/Hdgf/Smarca4/Stip1
darkturquoise	regulation of alpha beta T cell activation	6/284	95/15838	0.007237	Cd274/Cd55/Gata3/Hmgb1/Hsph1/Irf1
	positive regulation of interleukin 10 production	3/284	31/15838	0.017704	Cd274/Hmgb1/Hspd1
grey60	regulatory T cell differentiation	3/308	33/15838	0.025843	Foxp3/Fut7/Runx1
saddlebrown	negative regulation of map kinase activity	4/247	77/15838	0.032242	Aida/Dusp6/Gps1/Ptpn6
	negative regulation of T cell receptor signaling pathway	2/247	21/15838	0.041871	Phpt1/Ptpn6
	negative regulation of lymphocyte mediated immunity	3/247	53/15838	0.049639	Cd96/H2-Q7/Ptpn6
white	positive regulation of transforming growth factor beta production	2/244	19/15838	0.034024	Lgals9/Myb
	negative regulation of T cell receptor signaling pathway	2/244	21/15838	0.040955	Ptpn6/Ptprj

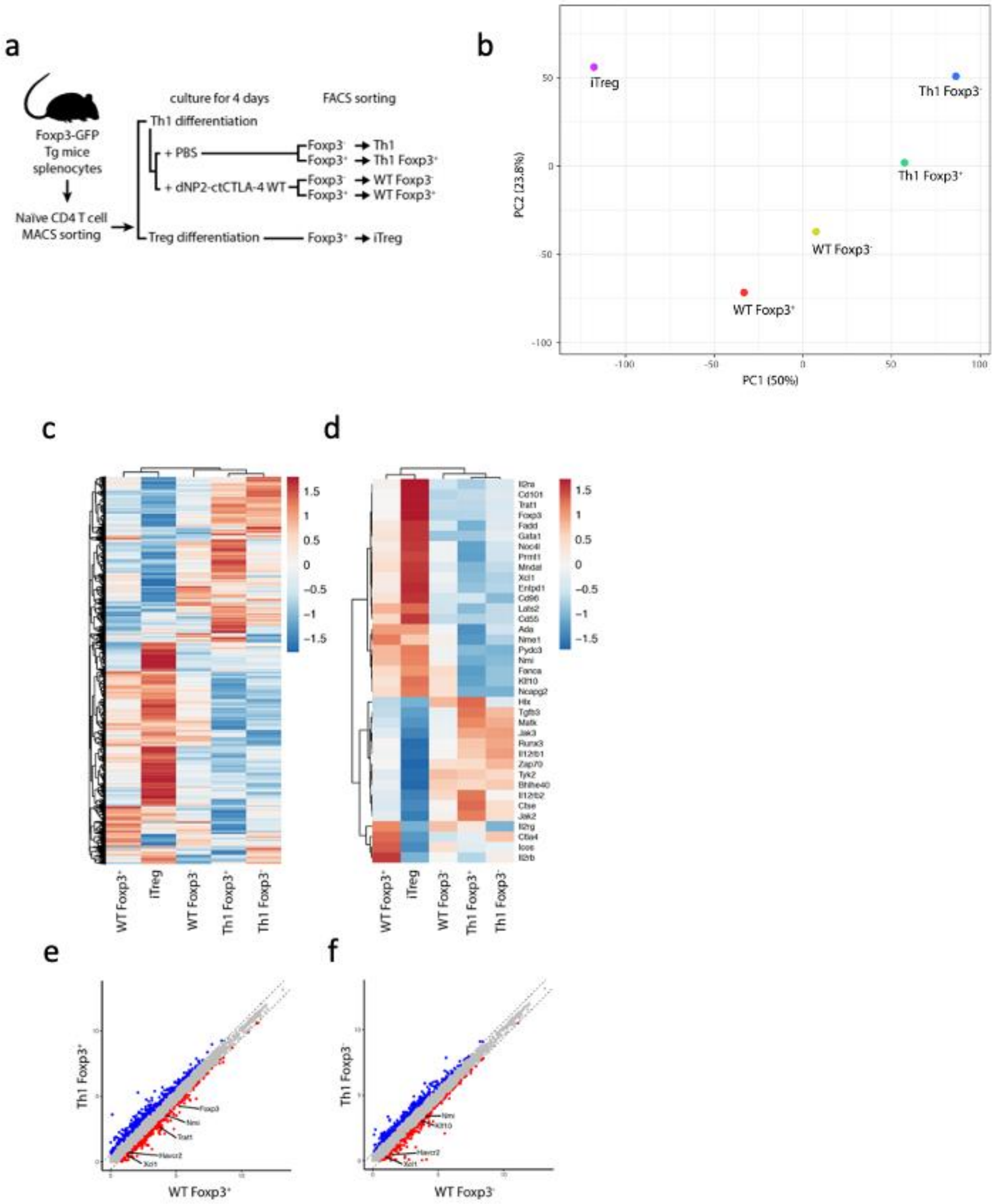

Figure S8. Transcriptome analysis of Foxp3$^+$ T cells induced by dNP2-ctCTLA-4 WT treatment under Th1 cell conditions reveals similarity to iTregs.

a) Scheme of RNAseq sample preparation. b) PCA plot of 12,874 expressed genes. c) Heatmap comparing differentially expressed genes (DEGs, fold change > 1.1) between Th1 cells and iTregs. d) Heatmap analysis of 37 genes of interest. e-f) Scatter plot indicating genes upregulated or downregulated over 1.5-fold in e) Th1 Foxp3$^+$ versus WT Foxp3$^+$ and f) Th1 Foxp3$^-$ versus WT Foxp3$^-$ cell populations.

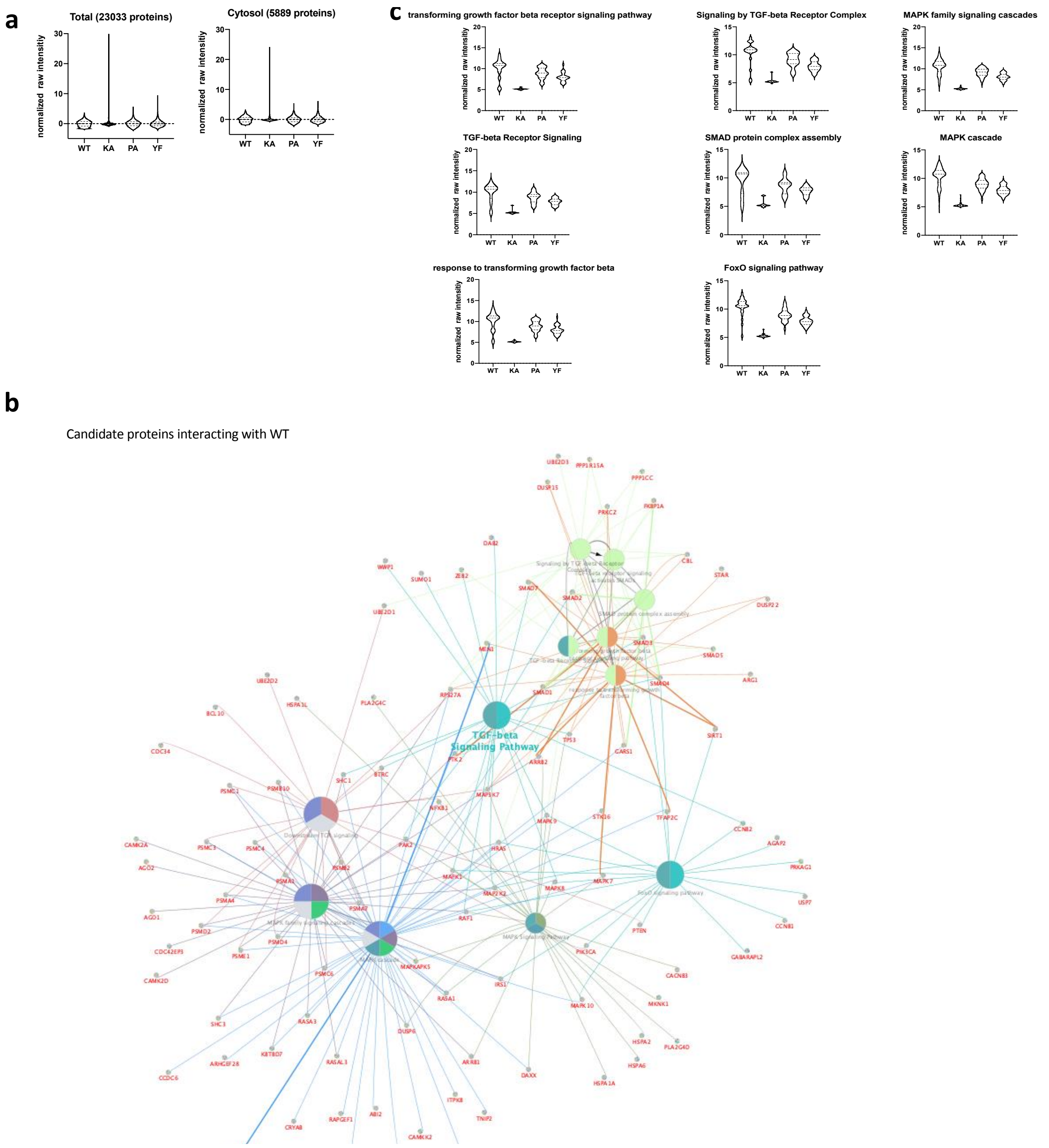

Figure S9. Huprot protein array analysis indicates that the lysine motif of dNP2-ctCTLA-4 is involved in TGF-β, SMAD, and MAPK signaling.

a) Violin plot of total or cytosolic targets identified using the Huprot proteome microarray. b) GO analysis of candidate proteins interacting with ctCTLA-4 WT peptide (z-score > 1, fold change > 2). c) Representative violin plot of GO terms of ctCTLA-4 WT peptide-interacting candidates.

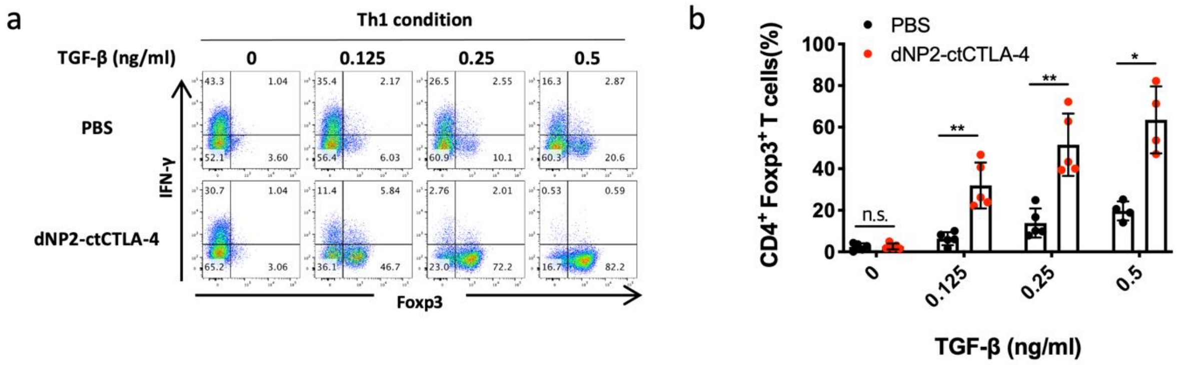

Figure S10. dNP2-ctCTLA-4 increases Foxp3^{+} T cells during Th1 differentiation TGF-β concentration-dependent manner.

Mouse naïve CD4 T cells were differentiated under Th1 conditions in the presence or absence of 2 μM of dNP2-ctCTLA-4. a) Representative dot plot and b) bar graph indicate that induction of Foxp3 expression by dNP2-ctCTLA-4 is dependent on TGF-β (n=4-5). Data are presented as mean ± S.D. Statistical significance was determined by Mann-Whitney test. n.s. = non-significant, *P<0.05, **P<0.01

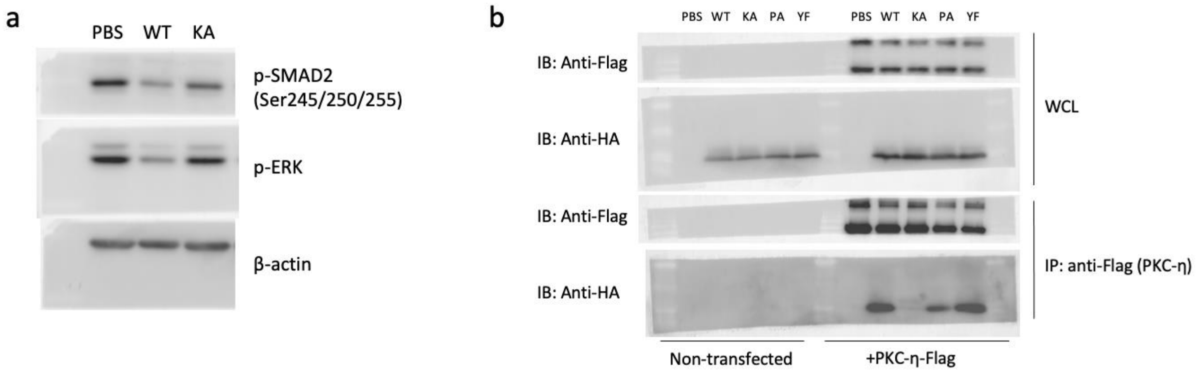

Figure S11. Uncropped full blot data.

a) Uncropped immunoblotting data of the p-Smad2 linker region; 2 μM of peptide-treated mouse splenocytes were stimulated with anti-CD3/28 and 5 ng/ml of TGF-β for 30 min. b) Uncropped immunoprecipitation and immunoblotting data of interaction between the lysine motif of CTLA-4 and PKC-η. HEK293 T cells were transfected with PKC-η-Flag-expressing vector for 48 h. After 48 h, cells were treated with 5 μM peptide for 2 h.

Table S2. Demographic information of the MS patients.

Demographic information of the MS patients from Seoul National University Hospital. MS patient PBMCs analyzed in this study were obtained from six donors.

Age	Sex	Disease	Disease stage	Current treatment	Any other comorbidities
32	F	MS	Remission	Tecfidera	None
39	F	MS	Remission	Tecfidera	None
33	F	MS	Remission	Tecfidera	None
31	M	MS	Remission	Tecfidera	None
35	M	MS	Remission	Fingolimod	None
26	F	MS	Remission	Aubagio	None

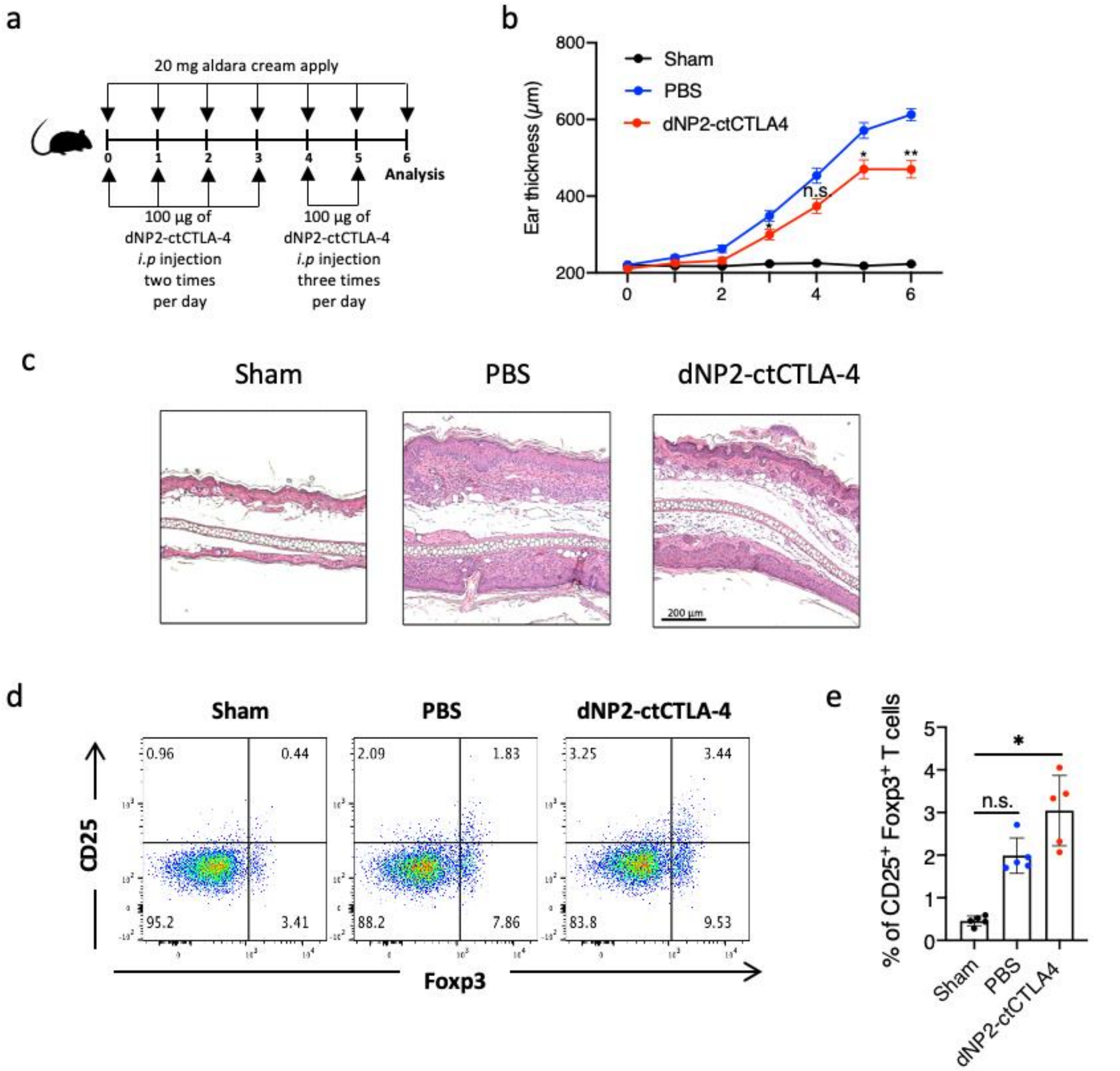

Figure S12. dNP2-ctCTLA-4 signaling peptide ameliorates imiquimod-induced psoriasis in mice with increased $CD4^+CD25^+Foxp3^+$ T cells.

a) Experimental scheme of the imiquimod-induced psoriasis mouse model. b) Ear thickness of mice with psoriasis-like skin inflammation (5 mice per group, representative data from two independent experiments). c) Histological analysis of ear-stained hematoxylin and eosin at day 6. d) Representative dot plot and e) bar graph indicate that $CD4^+$ $CD25^+$ $Foxp3^+$ population in the spleen. Data are presented as mean ± S.D. in b and e. Statistical significance was determined by Mann-Whitney test in b or Kruskal-Wallis test in e. n.s. = non-significant, **P*<0.05, ***P*<0.01.

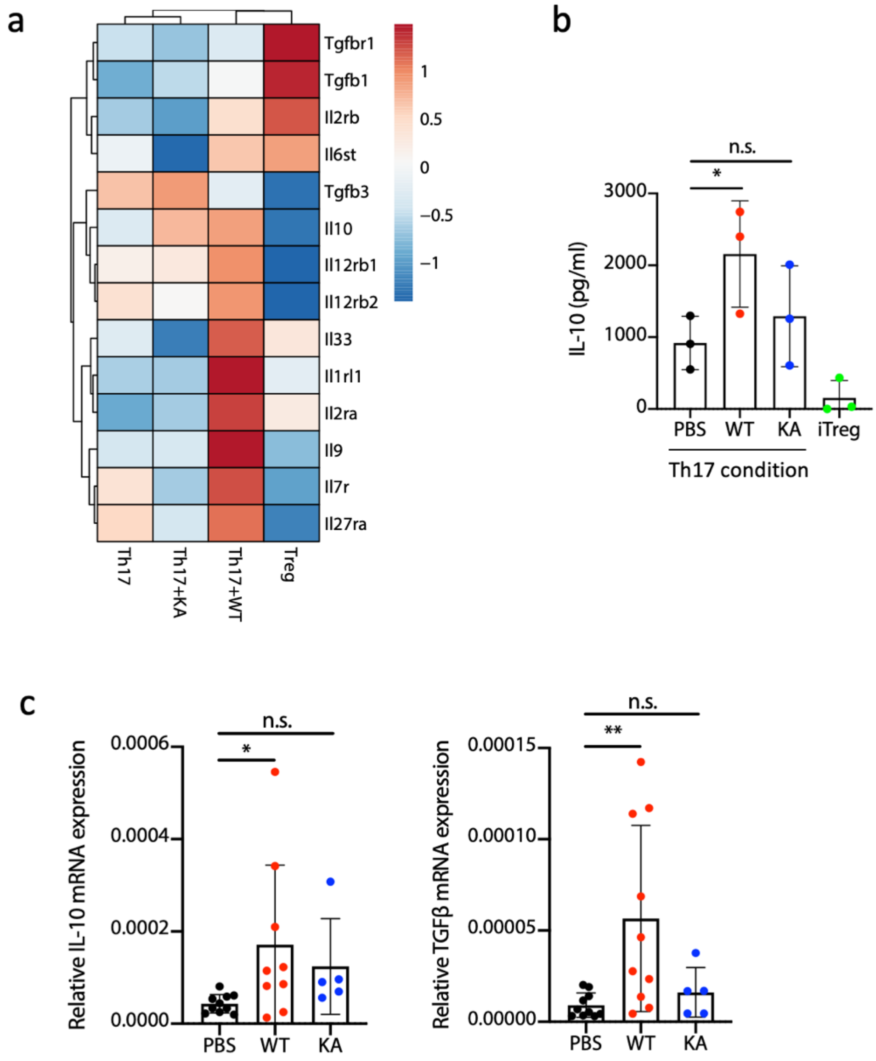

Figure S13. TGF-β and IL-10 expression levels were increased by CTLA-4 signaling peptide in Th17 differentiation conditions and spinal cords of EAE mice.

a) Heatmap of selected differentially expressed genes (DEGs) regarding Treg function. b) IL-10 ELISA under Th17 conditions with the peptides compared to iTreg *in vitro* (n=3). c) Relative IL-10 and TGF-β mRNA expression level in the spinal cord of EAE mice (5-10 mice per group, two independent experiments). Relative gene expression was normalized to the β2m gene. All data are presented as mean ± S.D. Statistical significance was determined by Mann-Whitney test in b or Kruskal-Wallis test in c. n.s. = non-significant, *$P<0.05$, **$P<0.01$.

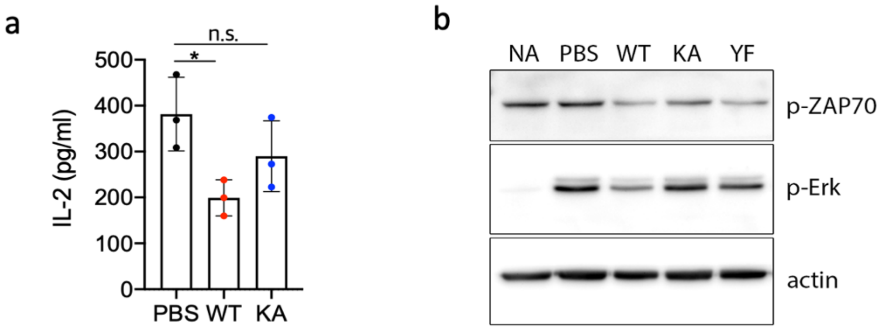

Figure S14. The dNP2-ctCTLA-4 inhibited TcR signaling and IL-2 secretion upon Th0 condition, but lysine mutant could not.

a) Naïve CD4 T cells were stimulated with anti-CD3 and anti-CD28 overnight in the presence of WT or KA peptide. At day 1, IL-2 level was analyzed by ELISA (n=3). b) Naïve CD4 T cells were incubated with 2 μM of each peptide for 1 hour and stimulated with anti-CD3/CD28 dynabeads for 30 min. The expression of p-ZAP70 and p-ERK were analyzed by immunoblotting. Data are presented as mean ± S.D. Statistical significance was determined by Mann-Whitney test in a. n.s. = non-significant, *P<0.05.

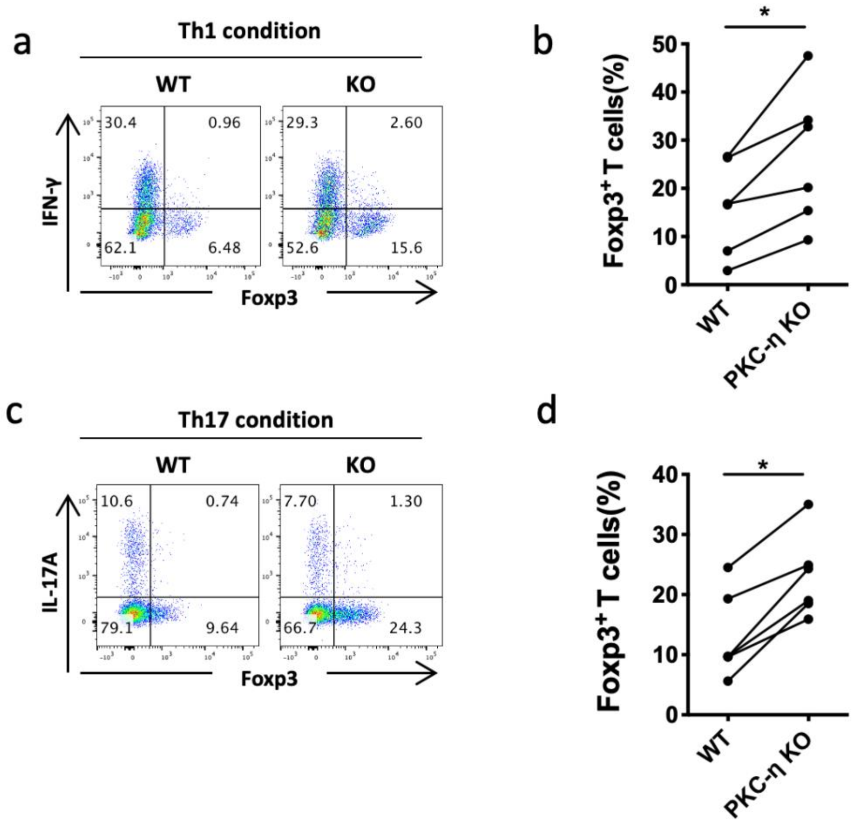

Figure S15. PKC-η KO T cells exhibit increased Foxp3 expression during Th1, Th17 differentiation.

Mouse naïve CD4 T cells from PKC-η KO mouse were differentiated a-b, under Th1 and c-d, Th17 differentiation conditions (n=6). a, c) Representative dot plot and b, d) bar graph indicate that Foxp3 expression increased in PKC-η KO mouse T cells compared to WT mice. Statistical significance was determined by Wilcoxon test. $^*P<0.05$

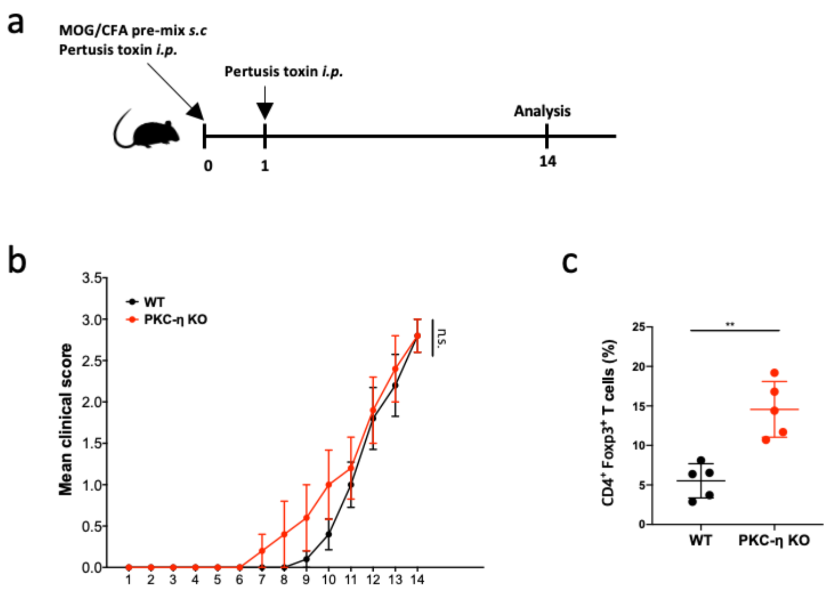

Figure S16. No difference on EAE progression between PKC-η KO and wild type mice.

a) Scheme of MOG-induced EAE model. b) Clinical score of EAE (5 mice per group). c) $CD4^+Foxp3^+$ T cells were analyzed at day 14. Data are presented as mean ± S.E.M in b and ± S.D. in c. Statistical significance was determined by Mann-Whitney test in b, c. n.s. = non-significant, $^{**}P$<0.01.
